# Synthesis, Characterization, and Roles of Vacancy Defects in Polymer and Graphitized Carbon Nitride Photocatalysts: A Comprehensive Review

**DOI:** 10.3390/polym17030334

**Published:** 2025-01-26

**Authors:** Arul Pundi, Chi-Jung Chang

**Affiliations:** Department of Chemical Engineering, Feng Chia University, 100, Wenhwa Road, Seatwen, Taichung 40724, Taiwan

**Keywords:** oxygen vacancies, conjugated polymer, polyimide, nitrogen vacancies, carbon vacancies, g-C_3_N_4_, role of vacancies, photocatalysis

## Abstract

Vacancy defect graphitic carbon nitride (g-C_3_N_4_) and conjugated polyimide (PI) polymer photocatalysts have become increasingly recognized as metal-free photocatalysts featuring an appropriate bandgap. The narrow absorption spectrum of visible light and the rapid recombination rate of the photoexcited charge carriers in PI polymers and g-C_3_N_4_ impede its photocatalytic performance. The presence of oxygen vacancies (OVs) in PI polymer photocatalysts, as well as nitrogen vacancies (NVs) and carbon vacancies (CVs) in g-C_3_N_4_, can significantly enhance the migration of photogenerated electrons. Adding vacancies to improve the electronic structure and band gap width can greatly enhance the photocatalytic efficiency of PI polymers and g-C_3_N_4_. Defect engineering is important for increasing the photocatalytic ability of PI-polymer and g-C_3_N_4_. There remains a notable absence of thorough review papers covering the synthesis, characterization, and applications of vacancy-rich PI-polymer and g-C_3_N_4_ in photocatalysis. This review paper examines the roles of OVs in PI-polymer, NVs, and CVs in g-C_3_N_4_ and thoroughly summarizes the preparation approaches employed before and after, as well as during polymerization. This review scrutinizes spectroscopic characterization techniques, such as EPR, XPS, PAS, XRD, FTIR, and NMR, for vacancy defect analysis. We also reviewed the role of vacancies, which include light absorption, photogenerated charge carrier separation, and transfer dynamics. This review could serve as a comprehensive understanding, a vacancy-engineered design framework, and a practical guide for synthesizing and characterizing.

## 1. Introduction

Semiconductor photocatalysis represents a significant technological advancement in tackling energy and environmental challenges. Polymer semiconductors have emerged as a novel category of heterogeneous photocatalysts due to their intrinsic benefits, including low cost, abundance in nature, straightforward synthesis, ease of functionalization, tunable optoelectronic properties at the molecular level, and high physicochemical stability [1,2,3,4]. Polymers can be used in photocatalysis applications, including covalent organic frameworks, covalent triazine frameworks, carbon nitrides, and conjugated polymers. The conjugated polymers, such as pyrene, carbazole, thiophene, porphyrin, hiadiazole, carbazole-anthraquinone, triphenylamine–anthracene, triphenyltriazine, pyrene–anthraquinone, tetrabenzophenazine, tetraphenyl-p-phenylenediamine, and imide-based polymers are reported in the photocatalysis applications [5,6].

Polyimide (PI)-based materials have widespread applications in various fields, including coating, separation, microelectronics, and aerospace industries [7]. In addition, PI was used in the electronics and optoelectronic sectors due to its remarkable chemical resistance and mechanical and thermal characteristics, which arise from its crystalline structure, conjugated framework, and π–π interactions [8,9,10,11,12]. PI has also attracted much attention in energy transformation and storage due to its exceptional redox activity and intricate charge transfer structures. Researchers are advancing the structural design of polyimide (PI) materials to enhance their photocatalytic and electrochemical properties. The modified PI structures have been developed as redox-active materials in photocatalysts, electrocatalysts, and energy storage devices such as batteries and supercapacitors [13]. The PI-based photocatalysts have recently gained significant interest in their photocatalytic applications due to their unpaired lone pairs of electrons in a π-conjugated system, structural stability, durability, and visible-light-driven photocatalytic reactivity. For example, Zhou et al. [14] synthesized polyimides that transition from dendritic to regular π-stacks via solvent-induced crystallization. The solvent selection was critical in the evolution of polymorphs, affecting chain diffusion and rearrangement through distinct interactions between the polymer and solvent. The PI-DMF hydrogen evolution rate was 16 times higher than that of PI-Toluene. It suggests that the DMF effectively facilitated ordered packing, highlighting the critical role of ordered π–π stacking in making polymer photocatalysts more effective [14].

**Figure 1:**
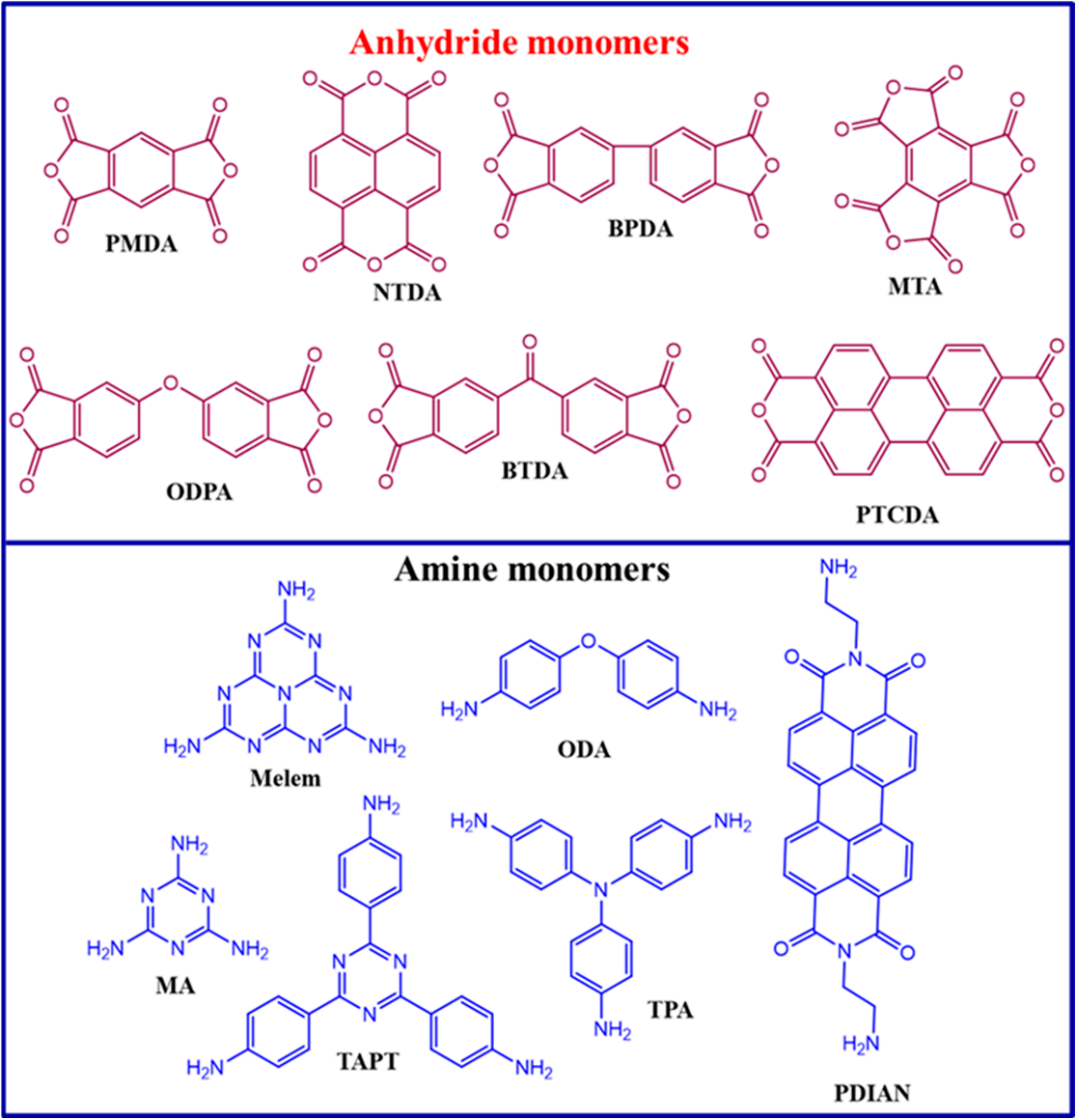
Chemical structures of the anhydride and amine monomers used in the synthesis of PI photocatalysts.

In conjugated polymers, combining electron-rich donor (D) and electron-deficient acceptor (A) units has proven to be a crucial tactic for optimizing energy band structures and improving charge separation. Polyimides (PIs) are a common D–A polymer. The selection of co-monomers significantly influences their photocatalytic performance and characteristics. To create PI-based photocatalysts, a variety of anhydride monomers are used, including pyromellitic dianhydride (PMDA), 3,3′,4,4′-Biphenyltetracarboxylic dianhydride (BPDA), 3,3′,4,4′-Benzophenone tetracarboxylic dianhydride (BTDA), Naphthalenetetracarboxylic dianhydride (NTDA), 4,4′-oxydiphthalic anhydride (ODPA), mellitic trianhydride (MTA), perylenetetracarboxylic dianhydride (PTCDA), and amine monomers such as melamine (MA), melem, tris(4-aminophenyl)amine, 4,4′-Oxydianiline (ODA), perylene 3,4,9,10-tetracarboxylic acid-bis-N, N′-p-aminophenyl diimide (PDIAN), and 2,4,6-tris(4-aminophenyl)-1,3,5-triazine (TAPT) (Figure 1) [5,15,16,17,18,19]. Anhydride monomers are essential in H_2_ production, CO_2_ reduction, and pollutant degradation using polyimide photocatalysts by adjusting the feed ratio of the amine and anhydride monomers. Anhydride-rich polyimide exhibits a lower valence band position and enhanced photooxidation capability, resulting in a greater tendency for water oxidation rather than water reduction when compared to amine-rich polyimide. This structure–activity relationship emphasizes the significance of redox energetics in the design of polymeric photocatalysts tailored for photoreactions [15,20,21].

Recently, researchers have introduced oxygen vacancies (OVs) in PI-based photocatalysts to enhance their photocatalytic activity. Introducing OVs in polyimide-based photocatalysts can improve photocatalytic performance by expanding the absorption of a range of light, acting as sites for molecular oxygen (O_2_) adsorption as well as activation, and improving the separation of photogenerated charge carriers [5]. For instance, incorporating oxygen vacancies into the PI framework can create mediated trap states beneath the conduction band (CB) of PI, resulting in improved visible light absorption [22]. Furthermore, surface oxygen vacancies can capture photogenerated electrons, which are subsequently transferred to adsorbed molecular oxygen (O_2_), resulting in the production of superoxide radicals (O_2_^−^), which is involved in the oxidative coupling of benzylamine [22]. The high concentration of OVs in the porous polyimide system facilitates the efficient separation of photogenerated electron–hole pairs, thereby enhancing the photocatalytic performance for the mineralization of methylene blue (MB) [23]. The bandgap energy of ~2.0 eV is ideal for visible light absorption. Fan et al. [18] prepared polyimide-based PTA-MA photocatalysts. By introducing the various concentrations of OVs during polycondensation, an ideal bandgap energy of ~1.37–2.0 eV is achieved. They also achieved a high conversion rate and selectivity for oxidative coupling of benzylamine and tetracycline (TC) degradation under visible light irradiation. Lan et al. [24] reported that the TFB-TPA photocatalyst containing many oxygen vacancies showed high oxygen adsorption in the reaction system. Meanwhile, the electronic localization surrounding the oxygen vacancy has the potential to facilitate the generation of free radicals, thereby offering additional active sites for catalytic reactions and enhancing catalytic activity in organic pollutant degradation. Wang et al. [25] noticed that the introduction of oxygen defects resulted in a broadening of the PI band gap and a notable blue shift of an absorption band edge. Nonetheless, both the separation and transport of photogenerated carriers were markedly improved. Consequently, the photocatalytic efficiency of the samples is effectively improved. Among the other photocatalysts, PI-NaHCO_3_ (1:4) demonstrated the most effective catalytic activity in photocatalytic chemical conversions (up to 99%).

Graphitic carbon nitride (g-C_3_N_4_) has become a metal-free photocatalyst due to its favorable electronic properties and stability [26,27,28]. The photocatalytic activity of g-C_3_N_4_ can be effectively improved by forming heterostructures or introducing surface defects [29,30,31]. The incorporation of carbon vacancies (CVs) can significantly alter the band structure, impacting wide-range light absorption and acting as an electron-trapping site, thereby enhancing the separation and transfer of photogenerated electron (*e*^−^) and hole (*h^+^*) pairs. This process minimizes recombination rates and increases the availability of more charge carriers in photocatalytic reactions. Research demonstrates that CVs can increase the density of states on the conduction band (CB) edge, thus facilitating electron transfer processes in photocatalytic reactions. Additionally, they provide more surface-active centers to improve O_2_ adsorption and lower the activation energy or overpotential associated with photoreduction reactions [30,31,32,33]. Incorporating carbon vacancies has been associated with improved photocatalytic performance in various applications [34,35,36,37,38].

Similarly to CVs, incorporating nitrogen vacancies in g-C_3_N_4_ enhances its photocatalytic activity by promoting electron migration and increasing the number of surface-active sites. This approach of synergistically modulating vacancy defects and one-dimensional nanostructures can greatly improve the photocatalytic performance of g-C_3_N_4_. Research indicates that the nitrogen vacancies (NVs) within tri-s-triazine units of g-C_3_N_4_ could modify the band structure, creating a new trap state and enhancing light absorption. The interplay between vacancy defects and one-dimensional nanostructures can significantly boost light harvesting capability, specific surface area, charge transfer, and diffusion processes in g-C_3_N_4_, enhancing photocatalytic performance [39,40,41,42].

The methods utilized for synthesizing carbon and nitrogen vacancies in g-C_3_N_4_ play a significant role in its photocatalytic performance. Researchers have been employing methods such as thermal treatment, hydrothermal synthesis, doping, and etching treatment to regulate the density and distribution of vacancies within the g-C_3_N_4_ structure. These methods can influence vacancy density, crystallinity, morphology, specific surface area (SSA), and porosity of g-C_3_N_4_. The capacity to customize these defects via synthesis techniques offers a means to enhance g-C_3_N_4_ photocatalysts in a wide range of applications [43,44,45,46,47,48,49,50,51,52].

**Figure 2:**
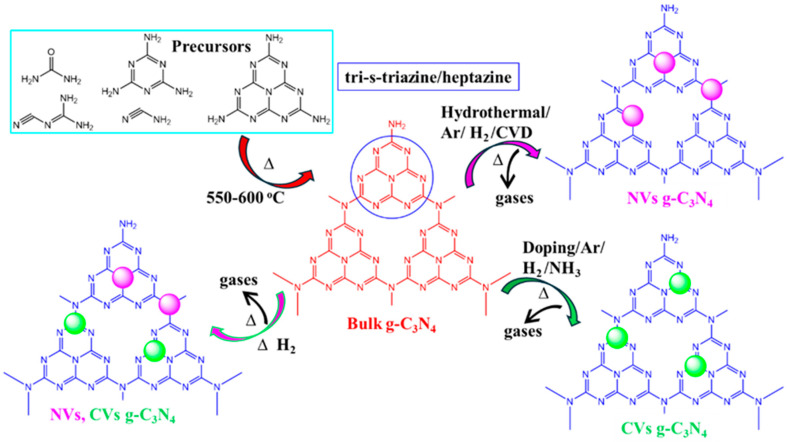
A schematic representation illustrating the insertion of NVs and CVs, or dual vacancies, in g-C_3_N_4_ (magenta and green color indicates the nitrogen and carbon vacancy. i.e., NVs and CVs, respectively).

Researchers have synthesized bulk g-C_3_N_4_ at elevated polycondensation temperatures using precursors like urea (CH_4_N_2_O), thiourea (CH_4_N_2_S), melamine (C_3_H_6_N_6_), cyanamide (CN_2_H_2_), and dicyanamide (C_2_N_4_H_4_). According to reports, all individual precursors produce a highly stable tri-s-triazine/heptazine (C_6_N_7_) ring structure after polycondensation in g-C_3_N_4_ [46,47,48,49,50,51,52,53,54]. Subsequently, the bulk g-C_3_N_4_ has been treated in pyrolysis/etching/doping, etc., to generate vacancies within the material. The release of gases such as SO_2_, NH_3_, CO_2_, and H_2_S, etc., during this process creates vacancies (CVs or NVs) and porous structures in g-C_3_N_4_ (Figure 2), which contributes to an enhancement in photocatalytic activity.

**Scheme 1:**
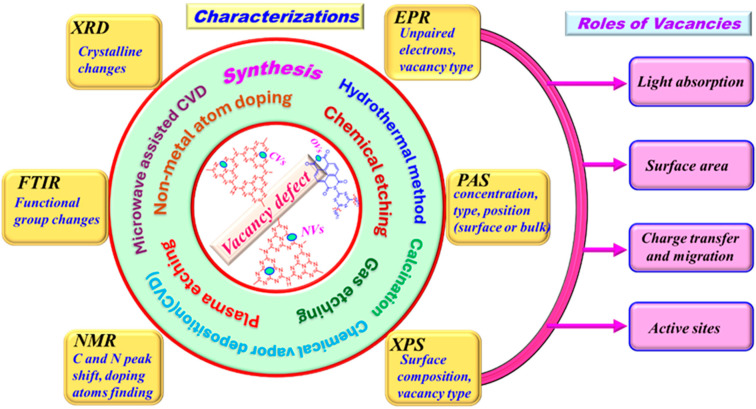
The formation methods, characterization, and roles of oxygen vacancies in PI-based photocatalysts, as well as carbon and nitrogen vacancies in g-C_3_N_4_ photocatalysts.

Covalent organic frameworks, covalent triazine frameworks, carbon nitrides, and conjugated polymers can be used in photocatalysis applications. Some reviewer papers report the defects of g-C_3_N_4_ photocatalysts. However, according to our literature review, the formation of defects in conjugated polymer photocatalysts and their influences on photocatalytic activity have not yet been reported in the review paper. In this review, we summarize (1) the formation methods, (2) characterization, and (3) roles of vacancies, focusing on oxygen vacancies (OVs) in PI-based photocatalysts, as well as carbon (CVs) and nitrogen vacancies (NVs) in g-C_3_N_4_ photocatalysts (Scheme 1). We examine several synthesis methods, including calcination, hydrothermal techniques, chemical vapor deposition, nonmetal atom doping, wet etching, and dry etching/plasma sintering processes. A comprehensive review of the characterization of vacancy-induced photocatalysts through various spectroscopic techniques has been conducted. This overview emphasizes their effectiveness in determining the types, positions, and densities of vacancies. Furthermore, we investigated the function of vacancies and their potential role in light absorption and harvesting, the role of active sites in aiding the adsorption of reactants, the role of allowed electron trapping activity, and photogenerated charge carrier dynamics, all of which are crucial for photocatalytic activity. This presents a comprehensive examination of different approaches and mechanistic evaluations to provide valuable information for future investigations into the design and development of PI-polymer and g-C_3_N_4_.

## 2. Construction and Control of Vacancies

### 2.1. Synthesis of Polymer Photocatalyst with Oxygen Vacancy

#### Thermal Treatment Method

**Figure 3:**
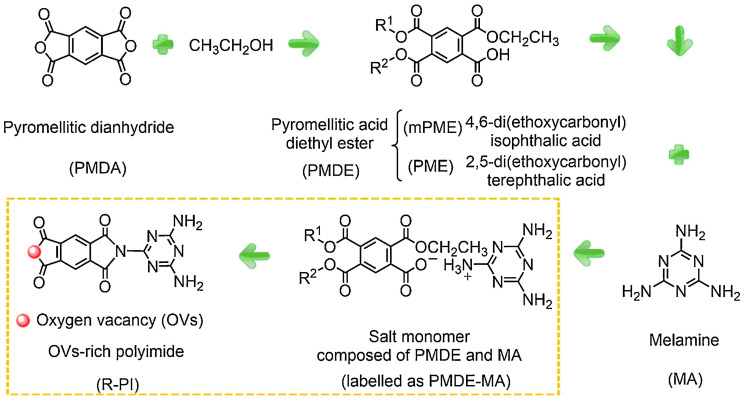
A schematic illustration of OVs R-PI synthesis using PMDE and MA (mPME: R^1^ = Et, R^2^ = H; PME: R^1^ = H, R^2^ = Et) [22].

Kong et al. [22] synthesized and reported conjugated polyimide (PI) photocatalysts with partially controlled oxygen vacancies (OVs) (O-PI and M-PI), deficient OVs (D-PI), and rich OVs (R-PI) through thermal solid-state polymerization at a reduced temperature (Figure 3). The only difference in the synthesis process of D-PI and R-PI is the PMDE-MA salt monomer. The D-PI is prepared by directly calcinating PDMA and MA with equal molar ratios. The synthesis of O-PI involved calcining the salt monomer in an oxygen atmosphere. However, M-PI was prepared using 50% oxygen/argon mixed gas during the calcination of the salt monomer. Compared with oxygen, the utilization of a 50% oxygen/argon atmosphere partially inhibits the generation of OVs in the PI. The OVs can be tuned by regulating oxygen concentrations. Due to its reduced band gap, the R-PI demonstrates extraordinary photocatalytic efficiency in selective benzylamine oxidation reactions under visible light. In addition, surface OVs act as outstanding active sites for the adsorption and activation of molecular oxygen (O_2_). Chemisorbed O_2_ creates a spatial channel that facilitates the delivery of photoexcited electrons to O_2_, thereby improving the separation efficiency of photogenerated charge carriers.

**Figure 4:**
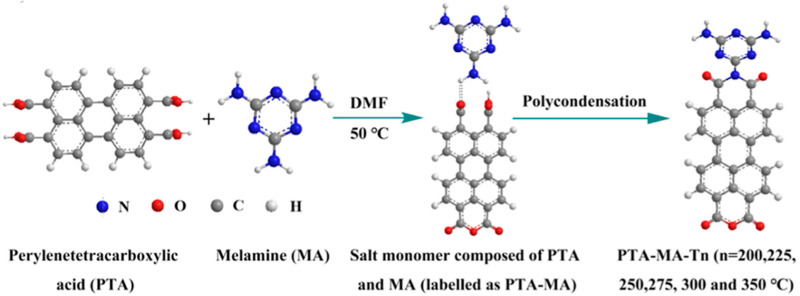
A schematic illustration of OVs polyimide PTA-MA-Tn synthesis at various temperatures (Tn) ranging from 200 °C to 350 °C [18].

The polycondensation temperature was varied to control the OV concentrations and the crystalline structures. Fan et al. [18] used solid-state polymer salt crystals from solid-state polymerization to create oxygen vacancies (OVs) in perylene polyimide (PTA-MA-Tn; Tn polymerization temperature) photocatalysts by combining salt crystals of perylene tetracarboxylic acid (PTA) and melamine monomer salt crystals (PTA-MA) (Figure 4). The X-ray diffraction (XRD), transmission electron microscopy (TEM), and electron paramagnetic resonance (EPR) revealed that PTA-MA-Tn produced at higher temperatures has a high degree of crystallinity and a significant signal owing to increased unpaired electron density. However, PTA-MA-Tn produced at a higher temperature, i.e., 300 °C (PTA-MA-300), demonstrated weaker photocatalytic activity than PTA-MA-Tn obtained at a lower temperature (PTA-MA-200), owing to poor separation of photogenerated electron-hole pairs and charge transfer. The photoluminescence and electron impedance spectra indicate that PTA-MA-200 has superior charge separation and electron transfer compared to PTA-MA-300. These results suggest that the increased crystallinity and dipole moment facilitated the migration of photogenerated electrons; however, further increasing the polycondensation temperatures led to enhanced π–π stacking and crystal sizes, which diminished photocurrent and intensified charge recombination. These findings suggest that the synthesis temperature of PTA-MA-Tn has a significant impact on its photocatalytic activity.

Wang et al. [25] synthesized a series of oxygen defect-rich (PI-NaHCO_3_) photocatalysts by using melamine and altering the molar ratio of pyrimellitic anhydride (PD) and foaming agent (NaHCO_3_) through thermal polymerization. After calcination, quenching the foam agent with dilute HCl (1 mole L^−1^) produces many bubbles that interfere with the polymerization of the polyimide precursor, resulting in the breakdown of the imide bond and the formation of defects. Varying the concentration of the foaming agent (NaHCO_3_) with polyimide has been used to control the oxygen vacancy defect in the PI-NaHCO_3_ photocatalyst. Low OVs in PI-NaHCO_3_ (1:1) and large OVs in PI-NaHCO_3_ (1:8) lead to decreased photocatalytic performances. When the defect concentration is either too low or too high, the edge of the PI-NaHCO_3_ light absorption band shifts towards blue, affecting its visible light absorption. While carrier separation has slightly improved, the combined effect results in reduced capability for photocatalytic hydrogen generation. The PI-NaHCO_3_ (1:4) photocatalyst demonstrated the highest hydrogen evolution activity and photocatalytic benzylamine oxidation. This is due to the synergistic impact of oxygen defects at optimum concentrations, which include increased specific surface area, modulated band structure, and enhanced carrier separation.

### 2.2. Synthesis of C_3_N_4_ with Carbon and Nitrogen Vacancy

#### 2.2.1. Hydrothermal

**Figure 5:**
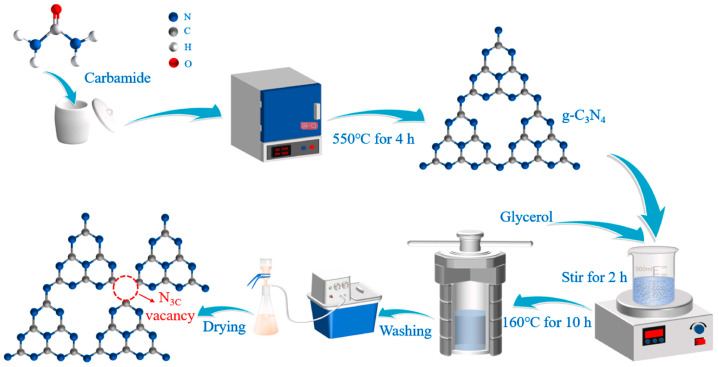
A schematic illustration of NVs synthesis at N_3_C sites in g-C_3_N_4_ [55].

Gong et al. [55] communicated the synthesis of g-C_3_N_4_ (CN) photocatalysts with many N_3_C vacancies and porous structures. As shown in Figure 5, they synthesized g-C_3_N_4_ by direct calcination using carbamide as a precursor. The CN was treated with water, glycerol, and a mixture of water and glycerol. Compared to glycerol, CN treated with water produced fewer vacancies. However, the mixed solution resulted in enhanced vacancy formation compared to that of glycerol. The existence of N_3_C vacancies (NVs) and the native absorption band of the π → π* electron transition generated a new absorption band for the n → π* electron transition n* in the region of 420–500 nm. Introducing N_3_C vacancies in CN can improve the light response, which is advantageous for the photocatalytic CO_2_ reduction activity.

Zhan et al. [56] reported strategies for synthesizing dual vacancies in g-C_3_N_4_ (V_C, N_-CN). V_C,N_-CN, which is co-modified with nitrogen-vacancy (V_N_) and carbon vacancy (V_C_), has been prepared through thermal polymerization combined with a hydrothermal process. Initially, they synthesized the nitrogen-vacancy g-C_3_N_4_ (V_N_-CN) using melamine and potassium tetrahydroborate (KBH_4_), calcining it at high temperatures. Later, the V_N_-CN was treated hydrothermally to obtain the V_C-N_-CN. According to the characterization results of V_C,N_-CN, they found that V_C_ was excessively generated only on the surface. In contrast, V_N_ was generated in the interior layers of the photocatalyst. Sample V_C,N_-CN exhibited outstanding photocatalytic properties, photodegrading 99.6% of TC in 3 min under visible light. Liang et al. [50] described the successful construction of a rod-shaped g-C_3_N_4_ photocatalyst with dual vacancies (CVs and NVs) and surface hydroxyl group modification using a simple sonication–hydrothermal method for Cr(VI) reduction in water. As the hydrothermal temperature rises from 120 to 180 °C, the structure changes from dense layer structures to nanorods with uniform pores due to the staking of sheet-like CN, leading to enhanced specific surface area.

#### 2.2.2. Calcination

Cheng et al. [57] synthesized a carbon vacancies g-C_3_N_4_ (TCN) photocatalyst through one-step thermal polymerization of the thiosemicarbazide (TSC) precursor. In g-C_3_N_4_ photocatalysts, the introduction of carbon vacancies is facilitated by the increased calcination temperature and self-generating gas environment, including N_2_H_4_, CS_2_, and H_2_S, during precursor (TSC) polymerization. The TCN-600 (calcinated at 600 °C) exhibits a new absorption band at approximately 460 nm in the absorption spectra. This band is attributed to the n-π* electron transition, which involves the lone pairs of edge nitrogen atoms in the heptazine units. It implies that carbon vacancies may lead to the construction of a partial asymmetric planar structure of TCN-600. Minh et al. [58] demonstrated how solvent-precursor interactions cause the formation of specific nitrogen vacancies in g-C_3_N_4_ structures, which benefits the piezo photocatalytic synthesis of H_2_O_2_ with high yields in clean water. They also established a relationship between crystalline defects and excited-charge kinetics, which has implications for solvent chemical structures and boiling temperatures. Li et al. [59] reported that synthesizing nitrogen vacancies g-C_3_N_4_ using a molten pretreatment technique resulted in a larger mesoporous structure and the development of nitrogen vacancies due to ammonia gas production. This approach increases the specific surface area of g-C_3_N_4_ 5.6 times (70.91 m^2^/g) compared to traditional calcined g-C_3_N_4_ (12.52 m^2^/g).

**Figure 6:**
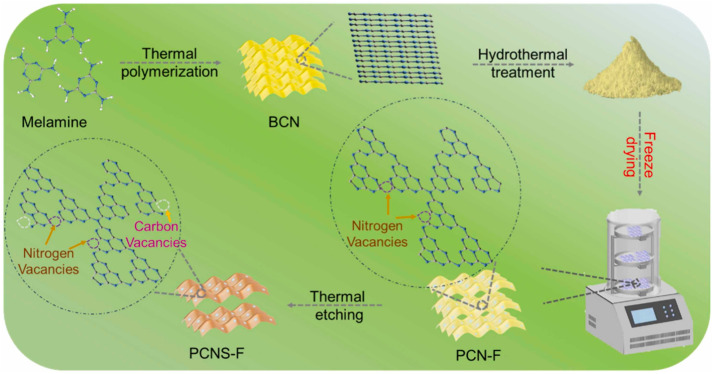
A schematic illustration of the synthesis of NVs PCN-F and dual vacancy PCNS-F photocatalysts [48].

Pang et al. [48] characterized and reported the significance of the drying process during the fabrication of vacancy-induced porous g-C_3_N_4_. Figure 6 illustrates the synthesis of vacancy-induced porous g-C_3_N_4_ NS (nanosheets). The bulk sample of g-C_3_N_4_ was prepared by traditional melamine calcination. Consequently, a template-free synthesis technique that included hydrothermal etching procedures was implemented to yield PCN-F (F—freeze drying) and PCN-T (T—dry at 65 °C) from bulk g-C_3_N_4_. Subsequently, the PCN-F and PCN-T were calcined to produce dual-vacancy PCNS-F (brown powder) and PCNS-T (white powder) photocatalysts. The only difference between PCNS-T and PCNS-F in the preparation process is the uncomplicated freeze-drying treatment, which occurs after hydrothermal treatment and before the thermal etching procedures. This treatment is critical for regulating the C/N ratio, N vacancies (NVs), and C defect (CVs) density. Surprisingly, the SSA of PCNS-F and PCN-F surpassed the PCNS-T and PCN-T, respectively. This suggests that the freeze-drying process contributes considerably to the expansion of the SSA and the improvement of the porous structures. 

#### 2.2.3. Chemical Vapor Deposition (CVD)

**Figure 7:**
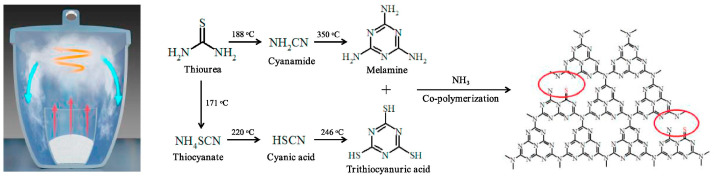
The formation mechanism of SCNNSs photocatalyst (red arrows indicates the vaporization precursors, red circle indicates carbon vacancies (CVs) [60].

Cao et al. [60] described an efficient method to create ultrathin sulfur-doped g-C_3_N_4_ porous nanosheets with carbon vacancies (SCNNS) for photocatalytic nitrogen fixation. SCNNS photocatalysts were created using gaseous copolymerization of melamine and trithiocyanuric acid in a self-generated NH_3_ environment. The NH_3_ atmosphere created carbon vacancies and nanosheets with hierarchical pore structures without significantly altering the chemical structure depicted in Figure 7.

#### 2.2.4. Microwave-Assisted CVD

**Figure 8:**
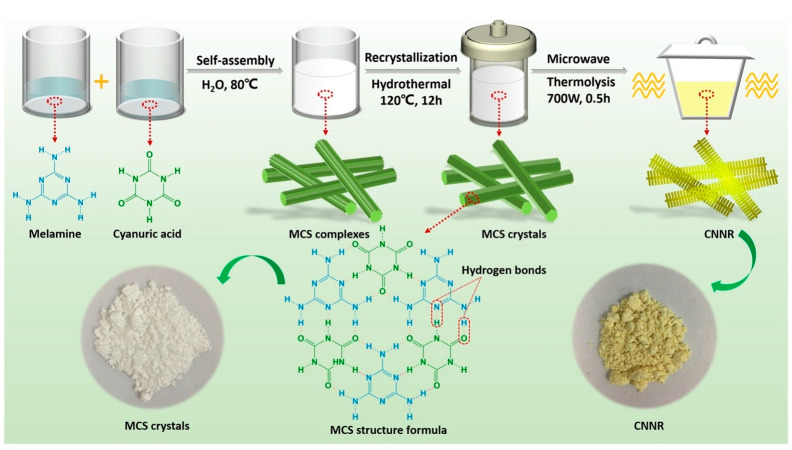
A schematic illustration depicting the fabrication of NVs CNNR via microwave-assisted CVD [61].

In a straightforward CVD method, the precursor was placed in a small crucible, positioned inside a large crucible, and sealed. These crucibles were heated at high temperatures to convert the precursor into a nitrogen/carbon vacancy g-C_3_N_4_. For example, Liu et al. [61] prepared a seaweed-like architecture of oxygen-doped nitrogen vacancy g-C_3_N_4_ nanoribbons (CNNR). They synthesized a melamine-cyanuric acid supramolecular (MCS) precursor, which was subsequently converted into CNNR through chemical vapor deposition (CVD) processes using microwave-assisted techniques (Figure 8). As the microwave thermolysis time increased, the samples transitioned in color from pale yellow to orange-yellow. The orange granules observed in the CNNR40 sample are indicative of nitrogen defects. The presence of nitrogen vacancies was confirmed by increased π-electron delocalization in the conjugated system of CN, as evidenced by the CNNR40 diffuse reflectance spectroscopy (DRS) spectrum.

**Figure 9:**
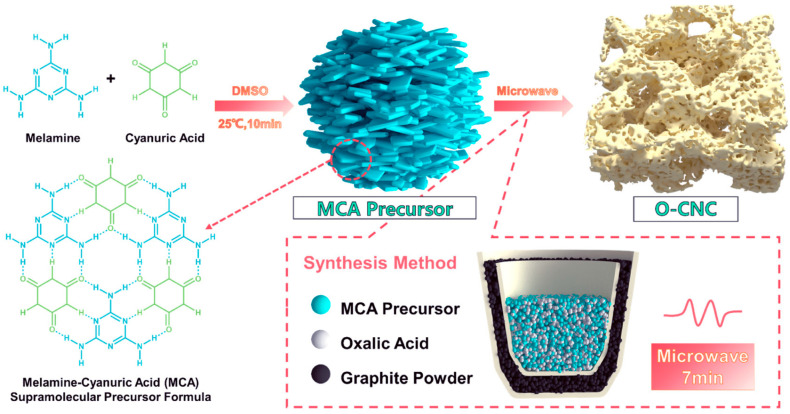
A schematic illustration of the synthesis of CVs O–CNC from the MCA precursor [62].

Xie et al. [62] used a microwave-assisted method to quickly prepare oxygen-doped carbon vacancy g-C_3_N_4_ (O-CNC) in just 7 min. The preparation of O-CNC involved using melamine-cyanuric acid supramolecular (MCA) precursors and oxalic acid as an oxygen dopant, as shown in Figure 9. They regulated carbon vacancies by adjusting the concentration of oxalic acid. The rate of photocatalytic H_2_O_2_ production by O-CNC is 2008.4 μmol h^−1^ g^−1^, which was 4 times greater than that of pristine bulk CN.

#### 2.2.5. Non-Metal Atom Doping

**Figure 10:**
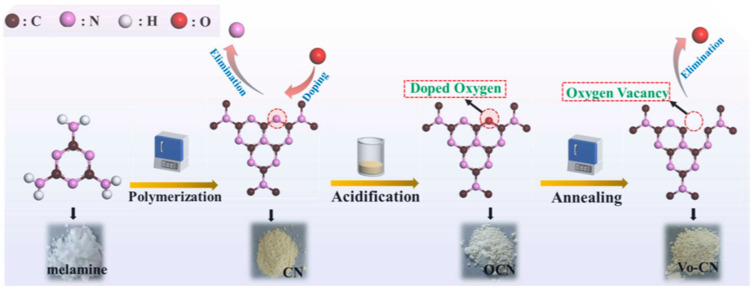
A schematic representation of the synthesis of V_O_-CN (heptazine unit) [63].

Zhang et al. [63] successfully made g-C_3_N_4_ with non-intrinsic oxygen vacancies (VO-CN) by using a series of steps for heating, acidifying, and heating-to-lower temperatures (Figure 10). Thermal polymerization synthesized pristine g-C_3_N_4_ (CN) from unprocessed melamine. The heptazine rings of the 3-s-triazine structural units of CN were doped with oxygen atoms through intense oxidation using a mixture of nitric acid and sulfuric acid. An innovative annealing process was employed to eliminate unstable doped oxygen in oxygen-doped g-C_3_N_4_ (OCN), forming oxygen vacancies in VO-CN.

Chen et al. [64] constructed a fluorine-coated carbon vacancy g-C_3_N_4_ (FCN-Cv) using urea and ammonium fluoride (NH_4_F). They investigated the photocatalytic ozonation performance by testing degrading sodium p-perfluorinated nonoxybenzenesulfonate (OBS) as a model pollutant. When heated to high temperatures, urea decomposes into ammonia and isocyanic acid ester. The isocyanic acid ester goes through rearrangement, polymerization, and zwitterionic polymerization. It causes polymeric nitrides to change to g-C_3_N_4_ (CN) through further annealing. They hydrothermally treated the bulk CN with NH_4_F, followed by calcination, which formed a fluorine coating and Cv vacancies in the bulk CN. By thermal treatment, Tian et al. [65] synthesized a porous powder of sulfur-doped carbon-vacancy honeycomb nanoflakes g-C_3_N_4_ (S-PDCN). They calcined the precursors (melamine, cyanuric acid, and thiourea) in a closed crucible at a high temperature (550 °C). Because of the lower decomposition temperature (320–360 °C), thiourea (TU) and melamine (MA) are very volatile in the high-temperature solid-state polymerization process. MA and TU thermally polymerize to generate g-C_3_N_4_, producing significant amounts of NH_2_ and CO_2_. Under the intensive effect of gases, g-C_3_N_4_ degrades rapidly, producing porous faulty nanosheets. S-PDCN demonstrates a photocatalytic H_2_ generation rate of 23.78 mmol g^−1^ h^−1^, 21.8 times higher than the bulk g-C_3_N_4_. Hong et al. [66] used high temperatures during the calcination process to dope and generate voids in g-C_3_N_4_. They constructed g-C_3_N_4_ doped with boron and nitrogen vacancy (NVCN) using sodium borohydride (NaBH_4_) to study ammonia photosynthesis from nitrate reduction. The nitrogen vacancy (NV) density in g-C_3_N_4_ was increased by progressively increasing the calcination temperature (400–500 °C). NaBH_4_ produces active H and B atoms that interact with nitrogen and carbon atoms in the g-C_3_N_4_ structure during calcination. Active hydrogen degrades an amino group (–NH_2_) to produce NH_3_ gas while also breaking the CN–C bond, resulting in NV. The method yields alkane gases and a cyano group (N≡C–).

#### 2.2.6. Etching Processes

##### Chemical Etching Method

Liu et al. [67] prepared nitrogen-defective g-C_3_N_4_ (UCN) using the reducing agent NaBH_4_ with different treatment periods, then calcinated at low temperatures for a short period of time. After etching with NaBH_4_, active hydrogen reduces the lattice N to NH_3_, followed by calcination, which creates nitrogen vacancies. They controlled nitrogen vacancies by adjusting the etching time. A longer etching duration could cause increased vacancies in the UCN due to N-to-NH_3_ conversion during NaBH_4_ treatment. However, Zhao et al. [68] reported that thermal treatment for 12 h at temperatures above 350 °C may introduce boron dopants into g-C_3_N_4_. Therefore, UCN was calcined for 15 min at 300 °C to prevent boron doping. Liang et al. [69] synthesized porous carbon nitride nanobelts with NVs (N_3_C sites) using a combination of sulfamic acid-induced supramolecular preassembly and thermal polycondensation. Oxoanions (SO_4_^2−^) trigger the spontaneous preassembly process of protonated melamine. In contrast to sulfuric acid, sulfamic acid has one donor site and three acceptor sites. This could change the structure of the supermolecule and the shape of the particles from irregular bulk to nanobelts. On the other hand, sulfamic acid in supramolecular structures may produce SO_2_ gas during thermal polycondensation, which speeds up the corrosion of structures and results in the formation of vacancies in the bulk CN. Here, they tuned the vacancy density in the bulk CN by adjusting the amount of NH_2_SO_3_H. As the amount of NH_2_SO_3_H increases, the vacancy density may increase due to the increased amount of SO_2_ gas produced during the polycondensation process. Gong et al. [35] synthesized a g-C_3_N_4_ with abundant carbon vacancies. During the calcination process, they controlled the carbon vacancies by adding different amounts of (NH_4_)_2_CO_3_ to the g-C_3_N_4_ aggregate (which was made from melamine). When heated to higher temperatures, the NH_3_ and CO_2_ generated by the decomposition of ammonium carbonate disrupt the molecular structure of g-C_3_N_4_, introducing carbon defects. The increasing percentages of (NH_4_)_2_CO_3_ created increased vacancies in g-C_3_N_4_. However, photocatalytic hydrogen production has declined because the increased number of vacancies often acts as a recombination center for e^–^/h^+^ pairs.

##### Gas Etching Method

Yan et al. [70] reported a sequence of g-C_3_N_4_ carbon vacancy photocatalysts (CN-x, where x = 1, 2, 3, 4, 5; x is the number of calcinations) and evaluated their photocatalytic hydrogen production activity. Here, they obtained CN-1 by directly calcinating the urea precursor in an atmosphere of N_2_. Furthermore, they calcined CN-1 at 400 °C for 60 min at a heating rate of 5 °C/min in an atmosphere of N_2_ to produce the carbon vacancy photocatalyst CN-2. The only difference between CN-3, CN-4, and CN-5 was the number of hot-cold cycles, which they used to regulate the density of carbon vacancies. As the number of hot-cold cycles increases, the vacancy content in CN-x exhibits a “volcano” tendency, as indicated by the intense CN-4 (fourth cycle) signal in the EPR signal.

Rao et al. [71] synthesized carbon vacancy CN (VCN) using CN etching with ammonia (NH_3_). The authors synthesized the bulk CN by heating the urea in air and then etching it in NH_3_ to obtain a VCN at high temperatures. When etched with NH_3_, CN gradually decomposes into a gaseous material. The released gas helps to disperse the agglomerate CN layer, resulting in several layers of porous nanowires with a large specific surface area. g-C_3_N_4_ etched in an NH_3_ environment outperforms pristine g-C_3_N_4_ for photocatalytic evolution of H_2_ under visible light illumination by 3.4 times (3304.5 μmol h^−1^.g^−1^).

Yue et al. [72] reported a carbon nitride (CN) photocatalyst with pyridinic nitrogen vacancies (N_2C_V-CN). Under natural sunlight and atmospheric oxygen, the N_2C_V-CN successfully induces a Fenton-like process for purifying organic contaminants. They prepared pyridinic nitrogen (N_2_C) vacancies by bombarding the bulk CN with high temperatures in the presence of Ar gas. During the photocatalytic degradation process, the pyridinic nitrogen vacancy in N_2C_V-CN generated lone paired and symmetry-unpaired electrons, which promotes the mobility of excitons and proton-coupled electron transfer to activate oxygen in hydrogen peroxide.

Liu et al. [33] proposed a modification of g-C_3_N_4_ using the steam activation-thermolysis method to control morphology, surface modification, and creating vacancies. The modification enhanced photocatalytic performance due to its high specific surface area, well-developed pore structure, and the synergistic effect of nitrogen vacancy defects and oxygen doping following steam activation. The steam activation time set was used to control the surface defects. Among the prepared photocatalysts, the ultrathin nanosheet shape CN-25, featuring more curled edges, facilitated higher photocatalytic activity.

Yang et al. [73] used (1) a hydrothermal process to prepare a self-assembled supermolecule nanorod (precursor) with a hexagonal columnar crystal structure from melamine and cyanuric acid; (2) porous nanotubes (TCN) prepared by calcination of the hexagonal precursor under Ar atmosphere; and (3) thin-wall nanotubes with carbon vacancy g-C_3_N_4_ (HTCN) prepared by calcination of TCN under 70 °C vapor using argon gas as a carrier gas. The HTCN synthesis method demonstrates that the hydrothermal process, followed by calcination under an Ar atmosphere, is critical in regulating the morphology. In contrast, calcination in an Ar carrier gas environment employing vapor is critical in producing vacancies.

**Figure 11:**
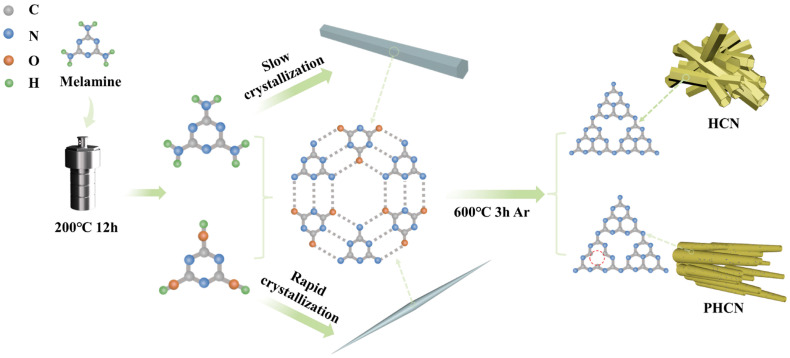
A schematic representation of the synthesis of HCN and PHCN (red circle indicates nitrogen vacancies (NVs) [74].

Han et al. [74], as illustrated in Figure 11, used a simple supramolecular self-assembly technique at lower temperatures to produce thin tube-wall porous hollow g-C_3_N_4_ (PHCN). They quickly chilled a hydrothermally reacted MA solution to generate a needle-shaped precursor (NP). The NP originates at low temperatures by supramolecular self-assembly, where fast crystallization restricts their longitudinal and lateral expansion, considerably lowering their size. It demonstrates that precursor morphology correlates with crystallization rate without the need for additional chemical treatments or modifications. Later, they used high-temperature pyrolysis to change the NP into nitrogen-vacancy g-C_3_N_4_ tubes with hollow, porous structures.

**Figure 12:**
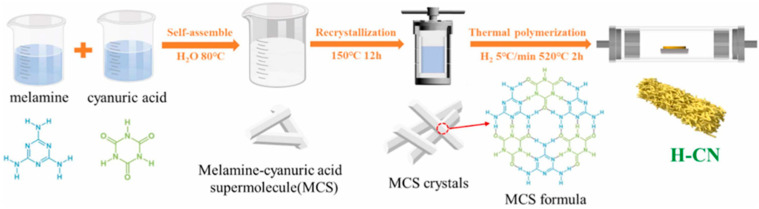
A schematic representation of the synthesis process for dual vacancy H-CN [75].

Zhang et al. [75] synthesized and reported dual-vacancy HCN using a hydrothermal method followed by calcination. The hydrothermal technique was used to create the MCS precursors from melamine and cyanuric acid. After the hydrothermal procedure, the MCS precursor was calcined at a high temperature (520 °C) for 120 min with an H_2_ flow (100 sccm) to produce carbon and nitrogen vacancies in HCN (Figure 12). H_2_ serves as a reducing agent. At higher temperatures, highly reactive H_2_ reacts more strongly with C and N atoms, transferring more energy. This strong interaction may simultaneously sputter C and N atoms on the CN surface, creating vacancies.

##### Plasma Treatment Method

Li et al. [76] used a dielectric barrier discharge (DBD) reactor and melamine precursor to synthesize the NVs g-C_3_N_4_. They utilized a plasma generator to generate a high voltage of approximately 10 kV while maintaining a steady supply of H_2_ at 40 mL/min. The discharge frequency was 10 kHz with a power input of 50 × 0.4 amps. They increased the plasma discharge time from 15 to 45 min to manage the NV concentration. They observed that when they synthesized g-C_3_N_4_ NVs with a 30-min plasma discharge duration, the concentration of NVs increased. They discovered that when they synthesized g-C_3_N_4_ NVs with a 45-min plasma discharge duration, the photocatalytic activity vanished because of the decreased surface area and reactive sites. Qu et al. [77] also employed the DBD plasma method to prepare NVs g-C_3_N_4_ by utilizing a melamine (MA) precursor, ensuring a plasma discharge duration of only 30 min. Plasma-induced NVs g-C_3_N_4_ demonstrates the maximum photocatalytic H_2_O_2_ production, about 5.2 mmol/L.

Zheng et al. [78] synthesized NVs-induced amorphous carbon nitride (ACN) via a one-step H_2_ plasma etching method, resulting in an exceptionally high specific surface area of approximately 405.76 m^2^g^−1^ and the incorporation of two N2C NVs within a singular CN matrix. They synthesized hierarchical NVs-induced ACN by subjecting the melamine-cyanuric acid supramolecular (MCS) precursor to H2 plasma at 150 Pa and a flow rate of 30 sccm at 400 °C for 90 min. Through XPS, EPR, FTIR, and 13C solid-state NMR analyses, along with first-principles computations, they determined that H_2_ plasma can incorporate two N2C-site NVs in a single CN matrix. 

There are various methods for forming vacancies in polymer photocatalysts, including calcination, thermal polymerization, hydrothermal, solvothermal, chemical vapor deposition, doping, etching, and plasma treatment. The advantages and disadvantages of these methods for the formation of vacancies are summarized in Table 1.

## 3. Characterization Investigations of Vacancies in Photocatalysts

### 3.1. Electron Paramagnetic Resonance Spectroscopy (EPR)

**Figure 13:**
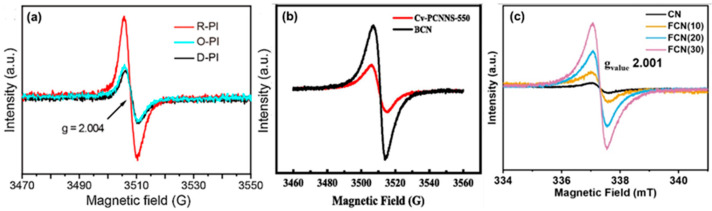
EPR spectrum of (**a**) D-PI, O-PI, and R-PI [22], (**b**) BCN and Cv-PCNNS-550 [32], (**c**) FCN and CN [80].

The electron spin resonance (ESR) can detect unpaired electrons. It is particularly responsive to paramagnetic materials such as vacancies. EPR can directly identify and quantify vacancies, especially oxygen/carbon/nitrogen vacancies in photocatalysts, by detecting unique signals corresponding to these defective sites. The expulsion of an atom in a lattice results in the formation of a single electron pair, which can be detected by EPR analysis. The existence and motion of unpaired electrons alter the signal intensity of EPR, allowing for an examination of various g values and signal strengths to gain insight into the type of vacancy flaws. EPR spectroscopy is critical for characterizing vacancy flaw types in g-C_3_N_4_. A g value of around ~2 demonstrates the existence of NVs in the defective g-C_3_N_4_, which can be recognized as a Lorentzian line [79]. Han et al. [74] synthesized NVs PHCN (conical porous hollow g-C_3_N_4_) and HCN (hexagonal tubes g-C_3_N_4_) displayed EPR signals at g = 2.002, which is attributable to carbon atoms with unpaired *e^−^* (electrons) on the aromatic heterocycles. The EPR signal intensity of PHCN is significantly greater than that of HCN, indicating that its backbone structure contains a greater number of carbon atoms with unpaired electrons. Kong et al. [22] employed EPR to investigate oxygen vacancies (OVs) in the samples of PI-based photocatalysts. Insufficient oxygen causes an unpaired electron to surrounding carbonyl carbon. They observed a single-feature EPR spectrum at g = 2.004 for polyimide, indicating the presence of unpaired electrons. The R-PI (OVs-rich) exhibited a more pronounced EPR signal than D-PI (OVs-deficient) and O-PI (OVs controlled by O_2_ gas) (Figure 13a). The concentration of OVs decreased after the molecule was heated in an oxygen atmosphere.

Li et al. [22] developed porous g-C_3_N_4_ nanosheets with carbon vacancies (Cv-PCNNS) as the photocatalysis-self-Fenton system. ESR results for C vacancies of bulk g-C_3_N_4_ (BCN) and Cv-PCNNS-550 exhibited a characteristic Lorentzian line from the unpaired electrons on the carbon atoms of the aromatic rings. However, the signal intensity of Cv-PCNNS-550 is lower. These data indicate the formation of CVs as the number of lone electron pairs decreases (Figure 13b). Similarly, Yang et al. [73] produced thin-wall nanotubes with carbon vacancy g-C_3_N_4_ (HTCN), which showed a decrease in peak intensity compared to TCNs (carbon nitride tubes), indicating the creation of carbon vacancies. As a result, the EPR signal drops dramatically as carbon atoms dissipate to produce carbon defects during vapor-assisted surface reconstruction. Preeyanghaa et al. [80] generated carbon-vacant CN (FCN), with the peak intensity increasing as the formalin level increased. The FCN has a considerable Lorentzian line intensity as the CV concentration increases. It is also worth noting that the number of self-doped oxygen atoms in the CN framework might cause the creation of an unpaired electron, resulting in an increase in peak intensity (Figure 13c).

### 3.2. Positron Annihilation Spectroscopy (PAS)

**Figure 14:**
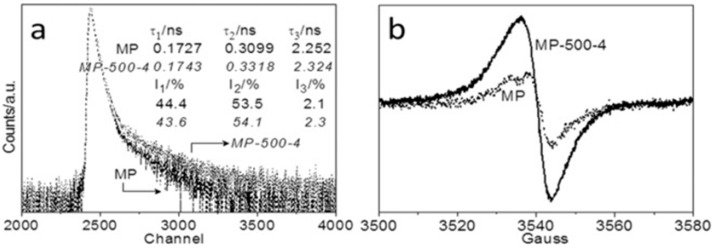
(**a**) PAS and (**b**) EPR spectra of the melon polymer matrix (MP) before and after steam etching under Ar carrier gas for 4 h at 500 °C (MP-500-4) [81].

PAS identifies vacancies, voids, and defects at the atomic scale. This method is particularly adept at identifying vacancy-type defects, offering detailed insights into the size, concentration, and nature of vacancies in the photocatalyst. PAS is a well-established approach for identifying vacancy-type defects in photocatalysts, providing detailed information about the size, concentration, environment, and type of vacancies. PAS can deliver three τ values as part of the characterization process, with different τ values corresponding to different defect sizes and locations. The τ_1_ value was attributed to the free annihilation of the positrons or tiny vacancies, while the τ_2_ value was assigned to the trapped positrons in large vacancies. The τ_3_ value refers to the annihilation of ortho-positronium atoms (triplet state of positronium) in the significant voids/interfaces. Minor defects exist in bulk samples, whereas significant faults are found primarily on the sample’s surface [81]. Consequently, as shown in Figure 14a, Yang et al. [81] noticed a significant change in the τ_2_ values after 4 h of melon polymer matrix (MP) steam etching, indicating the formation of more CVs on the photocatalyst surface. Compared to MP, the enhanced MP-500-4 EPR Lorentzian line intensity suggests the formation of unpaired electrons due to CVs formation (Figure 14b). The defect-engineered MP-TAP-CVs exhibited a 45-fold increase in photocatalytic carbon dioxide conversion activity over pristine MP.

**Figure 15:**
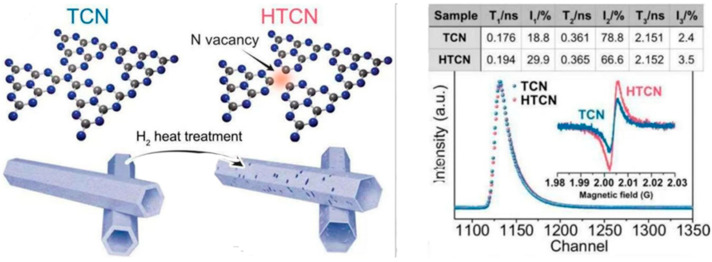
A schematic representation of the prepared route of HTCN and the PAS (Inlaying EPR spectra) of TCN and HTCN [34].

Gao et al. [82] found rich surface carbon defects in CN-C_V2_. The CN-C_V2_ sample showed an extensive increase in τ_2_ component concentration, possibly due to positron trapping by C-(N)_3_ defect sites. The relative concentration of CN-C_V2_ is 21.23 (*I*_2_*/I*_1_), which is much greater than that of CN (7.93), indicating a larger C-(N)_3_ defect concentration in CN-C_V2_. Yang et al. [83] used τ_1_ and τ_2_ to identify bulk and surface defects in PCN and PCN-50, respectively. They discovered that PCN-50 had a slightly longer τ_1_ lifetime (271 ps) than PCN (268 ps), indicating a low concentration of NVs in the bulk. However, a significant increase in τ_2_ from 365 ps (PCN) to 374 ps (PCN-50) indicates that NVs are widely distributed on the surface. Minh et al. [58] found that τ_1_ was associated with annihilations in perfect structures and monovacancies (NVs or CVs), but τ_2_ was associated with NVs + CVs clusters. They found that sample preparation significantly affected τ_1_, with CN having the lowest value (0.121 ns) and HCN and ICN having the highest values (0.152 ns and 0.154 ns, respectively), indicating a higher concentration of NVs. The slight change in τ_2_ between samples suggests that adding solvent during synthesis did not significantly influence the vacancy clusters. They reported that the generated clusters are likely NV-rich, such as 2CVs + 3NVs. Yang et al. [34] investigated TCN and HTCN using PAS, as shown in Figure 15. The data display three positron lifespan components (τ_1_, τ_2_, and τ_3_) and their intensities (*I*_1_, *I*_2_, and *I*_3_). HTCN’s τ_1_ and τ_2_ lifespans grow longer, to 0.194 ns and 0.365 ns, compared to TCN’s 0.176 ns and 0.361 ns. It shows that after H_2_ heat treatment, there are fewer nitrogen vacancies in the bulk phase and more on the surfaces of HTCN photocatalysts. On the other hand, HTCN showed a slightly longer τ_3_ (2.152 ns) than TCN (2.151 ns) and a higher *I_3_* (3.5% in HTCN vs. 2.4% in TCN), indicating more nanopores in HTCN. These results make HTCN suitable for catalytic reduction of imidacloprid within 20 min (100%).

### 3.3. X-Ray Photoelectron Spectroscopy (XPS)

**Figure 16:**
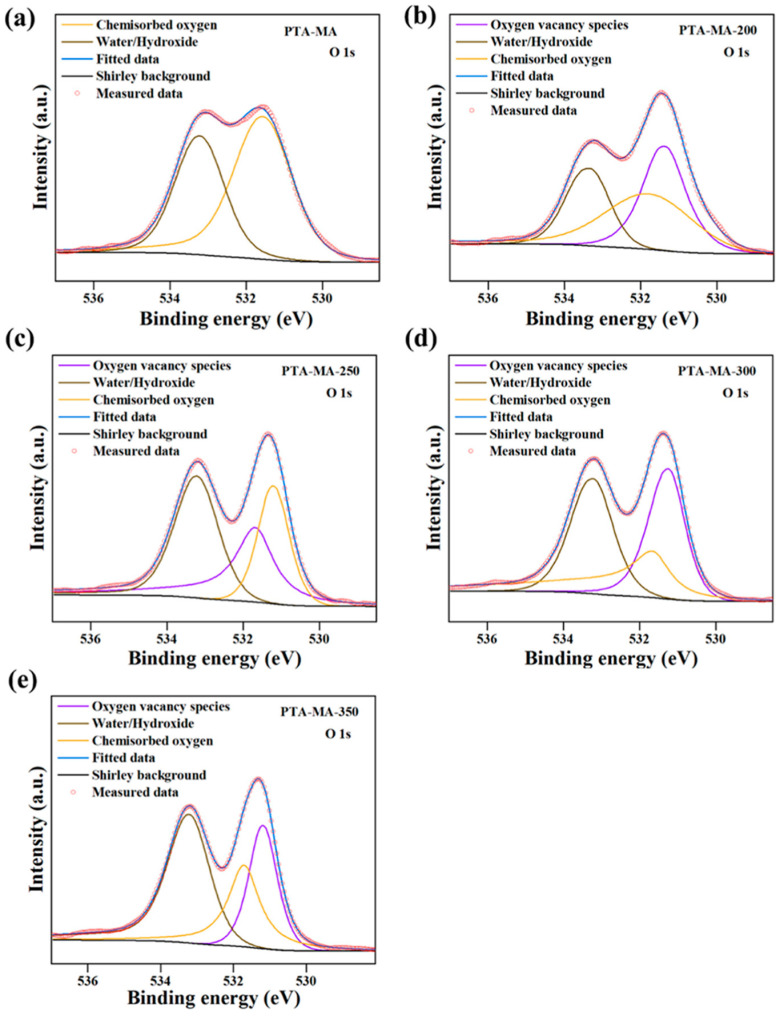
XPS spectra of O 1s, (**a**) PTA-MA monomer, and (**b**–**e**) PTA-MA-Tn prepared at different temperatures (Tn) ranging from 200 °C to 350 °C [18].

XPS is a commonly employed technique for investigating the types of vacancies present in g-CN and other materials. XPS examines the elemental composition of surfaces, providing valuable information about chemical environments and identifying different types of vacancies present in the g-CN. For instance, Fan et al. [18] reported the analysis of the OVs in PTA-MA-Tn photocatalysts using XPS (Figure 16). OVs play a significant role in affecting the redox capability of photocatalysts through their involvement in the photocatalytic reaction. They analyzed the O1s XPS spectra (Figure 4) and separated them into distinct peaks corresponding to OVs, water (H_2_O), and surface-adsorbed oxygen. The peak at 533.2 eV conceivably indicates physio-sorbed H_2_O, whereas 531.7 eV is associated with chemisorbed oxygen. The peak observed at 531.7 eV in PTA-MA samples signifies the presence of oxygen vacancies, as the elimination of H_2_O during polycondensation leads to a decrease in –OH groups. They found no OVs in the monomer salt (Figure 16a); however, the OV peaks increased with elevated polycondensation temperatures (Figure 16b–e).

**Figure 17:**
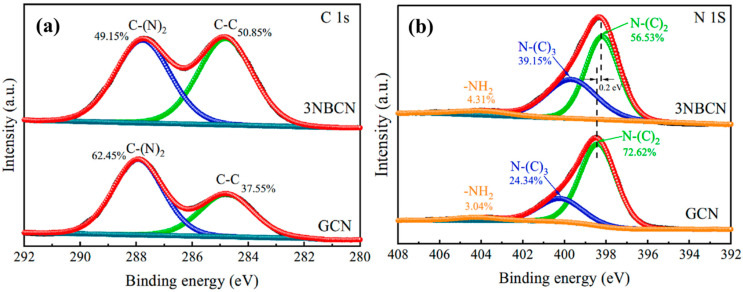
XPS spectra (**a**) C 1s (**b**), N 1s of GCN and CVs 3NBCN [36].

As depicted in Figure 17a, in the C 1s spectra, the C-(N)_2_ content in 3NBCN (49.15%) is significantly lower compared to GCN (62.45%). This disparity accounts for the decrease in carbon content in 3NBCN and provides evidence of the existence of CVs. CVs cause de-saturation of nitrogen atoms, resulting in a slightly broader peak that corresponds to –NH_2_ in 3NBCN (Figure 17b). Consequently, these nitrogen atoms bind to hydrogen atoms, leading to the formation of more –NH_2_, and significant changes are observed in the peak areas for N-(C)_3_ and N-(C)_2_. The increased CVs and intermolecular charge interactions might be responsible for this shift [36].

**Figure 18:**
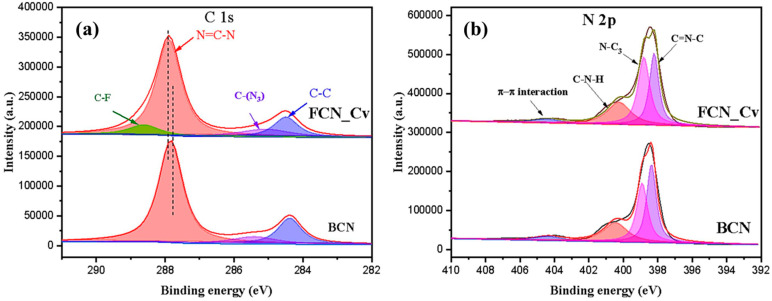
XPS spectrum of BCN and CVs FCN_Cv; (**a**) C 1s and (**b**) N 1s [64].

Chen et al. [64] observed a shift in the N=C–N peak to higher binding energy in the C 1s spectra (Figure 18a). This shift was attributed to the electron-withdrawing properties of fluorine. They also found that the percentage of hybridized N=C–N content of sp^2^ in FCN-Cv (68.3%) was slightly lower than in BCN (73.4%), suggesting the presence of CVs. Figure 18b displays the N 1s spectrum, which indicates that the percentages of N=C–N (32.3%) and N-(C)3 (21.6%) in FCN-Cv were lower compared to BCN (38.3% and 22.8%), respectively. These findings provide evidence of CVs within the tri-s-triazine units. The C–N–H group ratio in FCN-Cv (41.1%) was higher than BCN (33.3%) because of the generation of additional amino groups caused by adsorbed hydrogen atoms and unpaired nitrogen atoms during the formation of CVs.

Liao et al. [84] reported that a potential location for nitrogen vacancies appeared at position N1. The N–C3 ratio (0.59) shows an increase in NV-g-C_3_N_4_, while the C–N=C ratio (0.28) decreases compared to g-C_3_N_4_ (0.36 and 0.47), respectively. It suggests that the departure of nitrogen at the N1 position causes nitrogen vacancies. Yang et al. [85] revealed that the g-C_3_N_4_ structure favored vacancy formation more in N_2_C nitrogen atoms (two coordinated) than in N_3_C (three coordinated). The C_3_C peak in PNCN-2 exhibits a 0.2 eV shift towards higher energy, suggesting a deficiency of electrons. The loss of nitrogen atoms (N_2_C) is probably the cause, resulting in decreased valence electrons in carbon. After HPHT treatment, the N_2_C/N_3_C ratio decreased, indicating the possible presence of N_2_C vacancies. Gong et al. [55] reported that NV inclusion at N3C sites requires more energy. However, N_3_C site NVs form a more stable structure than N2C site NVs. After hydrothermal treatment of g-C_3_N_4_ with glycerol, they found that the ratio of N_2_C to N_3_C and –NH_X_ increased dramatically from 1.83 to 3.02 for GCN and 50CN, showing a decrease in N_3_C and –NH_X_ in 50CN. The N_2_C/N_3_C peak area ratio increases from 2.18 (GCN) to 3.78 (50CN), indicating extensive NVs at the N_3_C site. Li et al. [86] also constructed a vacancy at the N_3_C position and suggested that the N_3_C vacancies are more favorable in reducing NO to N_2_ and O_2_ than N_2_C.

### 3.4. Solid-State Cross-Polarized Magic-Angle Spinning NMR (SS CP-MAS NMR)

Researchers commonly use solid-state NMR spectroscopy to study material structure. New resonances emerge in defective g-C_3_N_4_ because of the differences in electrical and bonding environments created by the vacancies. It makes solid-state NMR useful for identifying vacancy defects. The g-C_3_N_4_ exhibits two significant resonance signals in ^13^C CP-MAS-NMR between ~150 to 170 ppm, which correspond to the C(1) and C(2) atoms in the tri-s-triazine rings. After introducing the vacancies, these peaks show changes in the peak shifting or intensity.

**Figure 19:**
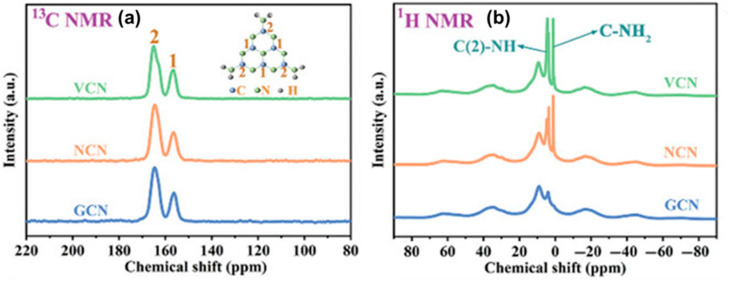
Solid-state (**a**) ^13^C NMR spectra and (**b**) ^1^H NMR spectra [87].

For instance, in the R-PI ^13^C NMR spectrum, Kong et al. [22] observed a new peak at 169 ppm, possibly due to the carbonyl carbon in a different environment, potentially linked to oxygen vacancies (OVs). O-PI does not have a peak at 169 ppm, and most peaks bear similarities with those of D-PI. The benzene carbon peak moving to 119 ppm in O-PI and R-PI shows that melt polymerization has improved polymer symmetry. The pronounced peak at 157 ppm further reinforces the high symmetry of O-PI. Liu et al. [87] investigated CVs-modified g-C_3_N_4_ (VCN) using the ^13^C CP-MAS-NMR. Two peaks at 157.2 and 165.6 ppm (Figure 19a) were observed corresponding to C(1) (N=C–N) and C(2) (N=C–N(NHx)), respectively. The C(2) to C(1) peak ratio in the defective material (VCN) rises dramatically, suggesting more CVs presence. Also, the ^1^H CP-MAS-NMR showed stronger signals at 1.03 ppm (C-NH_2_) and 3.61 ppm (C(2)-NH), which confirmed the CVs (Figure 19b).

**Figure 20:**
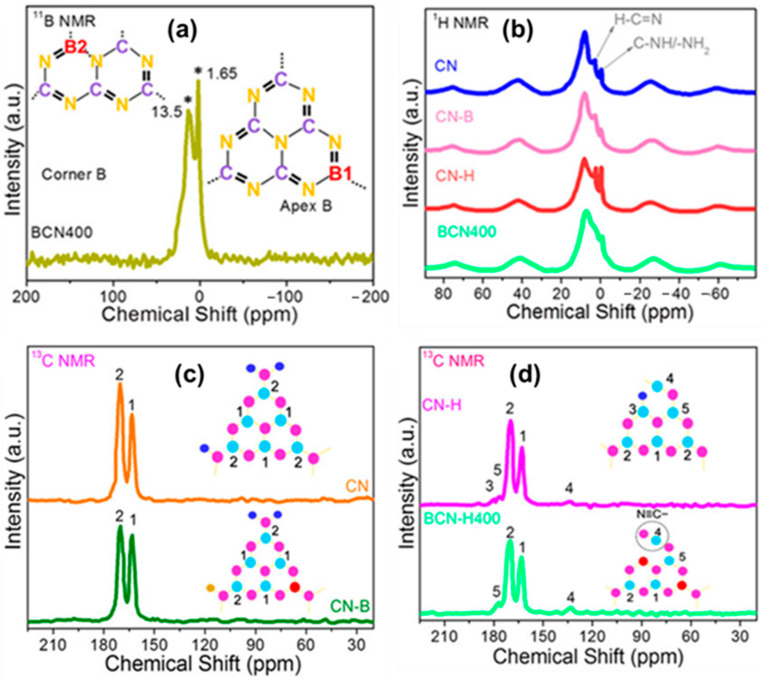
(**a**) Solid-state ^11^B NMR spectra of BCN400 (The * indicates the presence of Boron atoms instead of carbon atoms.), (**b**) solid-state ^1^H CP–MAS–NMR spectra, and (**c**,**d**) solid-state ^13^C NMR spectra of CN, CN–B, CN–H, and BCN400. Colors such as blue, sky blue, magenta, and red indicate hydrogen, carbon, nitrogen, and boron atoms (H, C, N, and B), respectively. The numbers indicate the positions of carbon atoms [89].

Jiang et al. [88] identified the nitrogen vacancy in the highly crystalline g-C_3_N_4_ nanosheets (hCN). After adding NVs through SPS, the solid-state ^13^C NMR spectrum of hCN revealed a new peak at 163.3 ppm. It might be due to an alteration in the chemical surroundings of C2 due to its sp^2^ hybridization. The absence of the nitrogen atom in the bridge position (N2) affects the chemical shift of C2, leading to an extra peak at 163.3 ppm. Khan et al. [89] investigated the molecular structure of boron-doped g-C_3_N_4_ with nitrogen defect samples using solid-state NMR, particularly ^11^B, ^1^H, and ^13^C CP-MAS. They meticulously examined the BCN400 (boron doped NVs CN prepared at 400 °C) sample and compared it to CN, CN–B (boron doped CN), and CN–H (nitrogen defect CN) to validate the boron doping and nitrogen defect (Figure 20). The ^11^B NMR spectra of BCN400 revealed two resonances at 1.65 and 13.50 ppm, confirming the presence of boron atoms at two distinct locations, B1 and B2, rather than carbon atoms (Figure 20a). A decrease in the intensity of N≡C–N(NHx) (C(2)) and N≡C–N (C(3)) can be seen in the ^1^H CP-MAS-NMR spectrum of BCN400, suggesting the presence of boron substitution (Figure 20b). The ^13^C CP-MAS NMR spectra of BCN400 show new peaks at 123.6 and 171.5 ppm due to structural alterations caused by boron doping and heat treatment (Figure 20c,d). ^13^C NMR of CN–B also revealed a decrease in the strength of the C(2) signal, confirming the influence on the structure. The treatment of the sample results in significant structural changes, including the substitution of carbon with boron and the introduction of nitrogen defects into the CN framework, as revealed by the NMR measurements. It influences the coordination of C–N–C and N–3C bridge constructions in the CN framework, exhibiting the dynamic chemical changes produced by boron doping and the resulting nitrogen deficiency.

**Figure 21:**
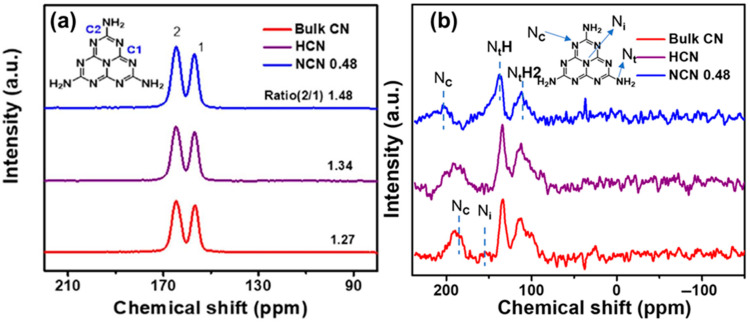
Solid-state (**a**) ^13^C NMR and (**b**) ^15^N MAS NMR spectra of Bulk CN, HCN, and NCN 0.48 [69].

Figure 21 presents a comparison of the b-CN, NCN, and HCN samples generated through the calcination of melamine, sulfamic acid-melamine, and sulfuric acid-melamine, respectively, to investigate the differences in NVs concentrations. In Figure 21a, for NCN 0.48, a greater C2/C1 intensity ratio (1.48) indicates nitrogen vacancies in the structure. The ^15^N MAS NMR spectra in Figure 21b show signals at 112.7, 133.7, 153.6, and 189.6 ppm for the N_t_H2, N_t_H, N_c_, and N_i_ atoms, respectively. The differences in N_c_ and N_t_ for NCN 0.48 corroborate with its distinct structure, supporting the existence of NVs [69]. 

### 3.5. Fourier Transform Infrared Spectroscopy (FTIR)

FTIR serves to identify functional groups and bonding characteristics. Vacancies may lead to new or altered vibrational modes in the infrared spectrum, signifying modifications in bonding conditions or new functional groups linked to the vacancies. Wang et al. [25] observed that the FTIR peak of PI-NaHCO_3_ showed increased absorption in the range of 3000–3400 cm^−1^ when compared to bare PI. This phenomenon can be correlated to adding the foaming agent NaHCO_3_, leading to the cleavage of the imide bond and the generation of a considerable amount of –NH2 groups, indicating the OVs in the PI-NaHCO_3_.

Adding ammonium acetate (CH_3_CO_2_NH_4_) causes a gradual drop in the peak intensity of g-C_3_N_4_-N_3_C-X samples at 890 cm^−1^ and 813 cm^−1^ [90]. This decrease may be attributed to the degradation of the N_3_C linkage sites of tri-s-triazine, reflecting the effective inclusion of NVs at N_3_C sites. More precisely, the faint peaks observed in g-C_3_N_4_-N_3_C-0.5 suggest that an increase in CH_3_CO_2_NH_4_ concentration will result in more nitrogen vacancies in the samples. As shown in the study conducted by Li et al. [91], the skeletal stretching model of Nv-M remains consistent throughout the evolutions compared to PCN (primitive g-C_3_N_4_), providing further evidence of the heptazine unit in the material’s structure. Furthermore, Li et al. [91] found two notable changes that become apparent with the increase in KOH. Firstly, the N–H stretched vibrations experience a gradual reduction in strength within the range of 3000 to 3350 cm^−1^. Furthermore, a new peak emerged at approximately 2174 cm^−1^, indicating a notable contribution from the asymmetric stretching vibrations of the cyano (–C≡N) groups. The peak intensity directly correlates with the increase in KOH usage, suggesting that the modification decreased the density of the N–H group and the addition of the –C≡N group. The –NH_x_ peak in the Cv-PCNNS-550 in the 3100 to 3400 cm^−1^ range was notably higher compared to other samples, suggesting a relatively higher concentration of amino groups and CVs [32]. The heptazine ring-breathing mode of FCN (20) undergoes a notable shift to 807 cm^−1^ when the formalin dosage is increased. This shift indicates that formalin dosage impacted the structural integrity of the heptazine framework of FCN due to CV formation [80]. Guo et al. [92] discovered that the fundamental structure of CN remained intact even after calcining the ball-milled precursor and introducing Br atoms with C, N-vacancies (CN_V_-mCN-Br). To validate the alterations in the CN_V_-mCN-Br, they conducted FTIR analysis. The peaks of C-N heterocycles in CN_V_-mCN-Br exhibit a lower intensity than CN and mCN. On the other hand, the CN_V_-mCN-Br sample exhibits a greater intensity of N–H-related bands than the CN and mCN samples. A new peak in the fingerprint region indicates C–Br (594 cm^−1^) stretching vibrations. The results suggest that the changes in the C–N lattice structure, the increase in N–H groups, and the introduction of Br doping in the CN_V_-mCN-Br framework contribute to the formation of CVs and NVs.

### 3.6. X-Ray Diffraction (XRD)

XRD is applied to examine the crystal structure, phase composition, and crystallite size of photocatalysts. Alterations in peak positions, intensities, and widths in the XRD pattern may signify the existence of vacancies, which can lead to lattice distortions and influence the periodicity of the crystal structure. Researchers commonly employ the technique of XRD to recognize and differentiate the presence of vacancies in g-C_3_N_4_ photocatalysts. The presence of vacancies within the material’s tri-s-triazine framework disrupts its periodic structure. Additionally, a significant concentration of vacancies affects the interlayer distance and the overall crystallinity of the material [87,93,94]. Wang et al. [25] reported that after treating PI with an increasing molar ratio of PD to NaHCO_3_, the peak intensity consistently decreased, indicating reduced crystallinity due to the introduction of OVs disrupting the imide bonds. Additionally, they observed that the diffraction peaks of PI-NaHCO_3_ samples exhibited a slightly blue shift compared to PI, potentially linked to the increase in oxygen vacancies induced by NaHCO_3_.

**Figure 22:**
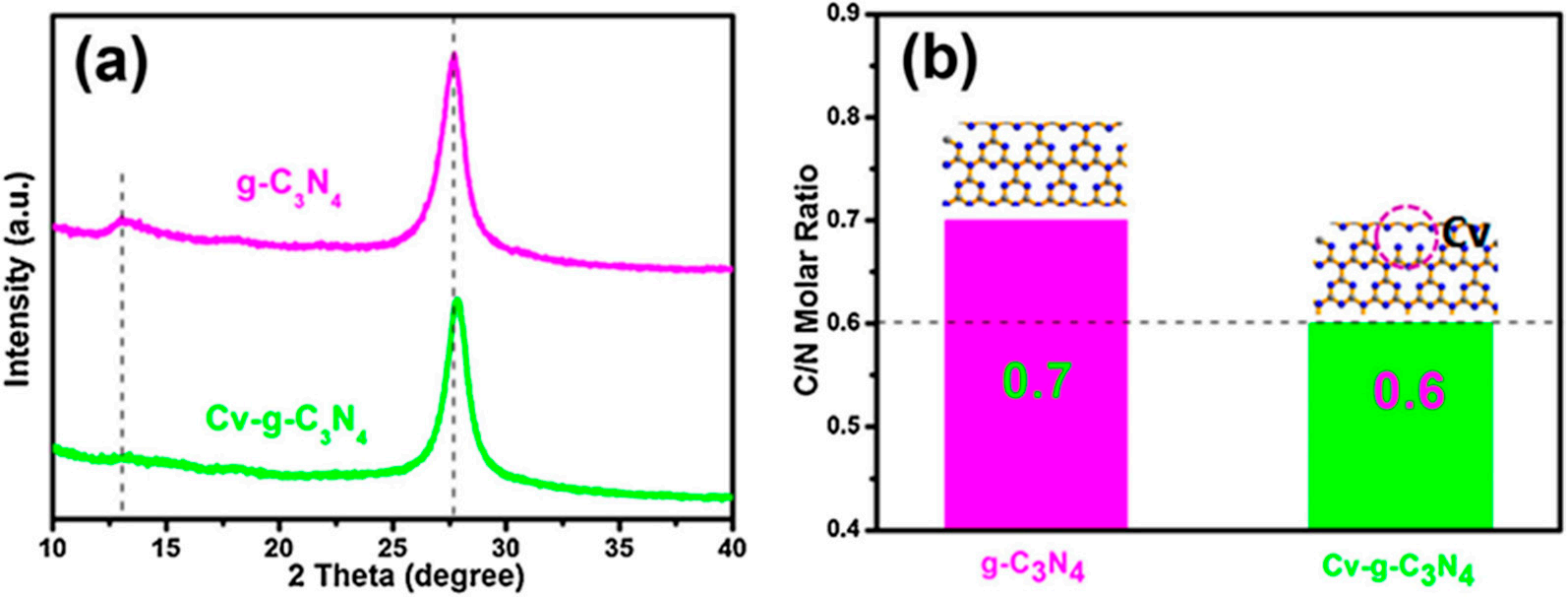
(**a**) XRD patterns of g-C_3_N_4_ and Cv-g-C_3_N_4_; and (**b**) C/N molar ratio in the g-C_3_N_4_ and Cv-g-C_3_N_4_ [93].

Wang et al. [95] investigated the XRD patterns of all photocatalysts to determine how oxalyl dihydrazide (ODH) affects crystal structure. Two peaks in XRD patterns were identified with unchanging positions: the smaller peak at 13.1° represents tri-s-triazine units (100) in-plane packing. In comparison, the stronger peak at 27.6° (002) is caused by π–π interlayer stacking along the c-axis. ODH-CNX had a stronger 27.6° peak than CN, indicating higher crystallinity. They fitted the 27.6° peak profile with Gauss and found that ODH-CN2 had the narrowest full width at half maximum (FWHM), indicating the highest crystallinity. The heat released when N_2_H_4_ breaks down during calcination probably helped precursor polymerization, which increased crystallinity and exciton dissociation, charge transfer, and photocatalytic activity. However, beyond 0.2 g, ODH expanded the FWHM, possibly due to a thinner layered structure and lower crystallinity. The decreasing crystallinity is due to the formation of highly rich N vacancies in g-CN, which disfavors photocatalytic activity. Zhang et al. [96] observed that the g-C_3_N_4_-V XRD peak, which was made by two-step calcination, was weaker than the bulk C_3_N_4_ peak. Additionally, they found an increase in the half-width of the peak, indicating a decrease in the crystallinity of g-C_3_N_4_. The existence of vacancies in the g-C_3_N_4_ skeleton may cause this decrease in crystallinity. Furthermore, the (0 0 2) peak exhibited a slight shift, suggesting that the inter-layer repulsion is reduced due to the two-step-thermal oxidation treatment. Li et al. [91] reported that pristine PCN material exhibited a prominent diffraction peak at 2θ = 27.3° (002) and a less intense peak at 12.7° (100). However, in defective Nv-M, the intensity of these peaks decreases due to the disrupted structure. Furthermore, they observed a shift in the (002) peak when the stacking distance between the nanosheets decreases. As shown in Figure 22a, Li et al. [93] found that the Cv-g-C_3_N_4_ peak was weaker and moved toward a higher 2θ value, suggesting that argon gas heat treatment may damage the g-C_3_N_4_ crystallinity, which indicates the formation of defects. They used elemental analysis in addition to XRD data to determine the generation of CVs in g-C_3_N_4_. The reduced C/N molar ratios in Cv-g-C_3_N_4_ indicate the presence of CVs (Figure 22b).

## 4. Roles of Vacancies

The photocatalytic activity can be increased by enhancing the light-harvesting, active site, surface area, and charge transfer of photocatalysts [97,98,99,100,101,102]. The formation of vacancies in photocatalysts can play the following roles, leading to improved photocatalytic activity.

### 4.1. Influence on Band Structure and Light Absorption

In addition to surface texture-induced light-trapping and increasing the conjugation length of the molecular chain [103,104,105,106,107], the light-harvesting could be improved by introducing nitrogen vacancies and carbon vacancies [36,90]. The vacancy-rich g-C_3_N_4_ efficiently harvests light through multiple reflections and scattering, facilitated by the porous structure formed during synthesis. This results in reduced transparency, improved light absorption, and decreased surface diffusion distances, ultimately facilitating charge carrier separation. When a material is exposed to incoming light, photons may be absorbed if its band gap fits with the photon’s energy. g-C_3_N_4_ has a relatively low bandgap of around 2.7 eV, making it ideal for absorbing photon energy in the visible light spectrum [108,109]. Introducing vacancies can alter the band structure, often reducing the band gap. The inclusion of NVs moves the conduction band downward, while the CVs shift the valence band upward [109]. These alterations are critical to the photocatalytic activity of defective g-C_3_N_4_ in fields such as H_2_ generation [110,111], CO_2_ reduction [112], environmental remediation [113], etc. The absorption characteristics of the material are better aligned with visible light by adjusting the band gap using vacancies.

The presence of NVs and π-conjugated structures in g-C_3_N_4_ alters the electronic band structure, resulting in defective midgap states in between the bandgap to improve light absorption [114]. Compared to the original g-C_3_N_4_ (CN), the nitrogen-vacancy g-C_3_N_4_ (Nv-RCN) exhibited a noticeable shift towards longer wavelengths in its absorption edges, suggesting a much-improved ability to absorb light. The band gap energies (Eg) for CN and Nv-RCN are 2.81 eV and 2.11 eV, respectively. Nv-RCN demonstrated an impressive propylparaben elimination effectiveness of 94.3%, surpassing CN by 3.37 times [41]. Xue et al. [90] found that NVs in the N_3_C sites significantly altered the energy bands of g-C_3_N_4_. The 400–800 nm light absorption intensity increases as the nitrogen vacancy increases compared to that of pristine g-C_3_N_4_. The presence of nitrogen vacancies explains the red shift and enhanced light absorption intensity observed in g-C_3_N_4_-N_3_C. N_3_C vacancies in g-C_3_N_4_ preserve the molecular structure within the plane. Simultaneously, the N_3_C vacancies enhance the light absorption intensity and boost the charge transfer efficiency. It is attributed to the formation of an intermediate bandgap triggered by NVs (N_3_C sites).

Changing the nitrogen defect density leads to variation in the bandgap. Liu et al. [67] modified a band gap by enhancing the nitrogen vacancy density by treating bulk g-C_3_N_4_ (UCN) with NaBH_4_ over various time intervals. The bandgap of the pristine UCN measures 2.71 eV. After a 30-min treatment with NaBH_4_, it decreases to 2.62 eV (UCN-0.5 h). After 60 min, it is reduced further to 2.59 eV (UCN-1 h). This indicates a narrowing of the bandgap with an increase in nitrogen density. However, additional treatment for 120 min led to an increase in the bandgap to 2.65 eV (UCN-2 h) alongside rising nitrogen densities. It was observed that UCN-1 h exhibited superior photocatalytic activity compared to pristine UCN, UCN-0.5 h, and UCN-2 h. The UCN-2 h exhibited subpar photocatalytic performance, as the rise in nitrogen vacancies creates new recombination centers for photogenerated charge carriers, leading to inadequate separation and consequently diminishing activity. Huang et al. [115] reported that the indirect bandgap of g-C_3_N_4_ decreased from 2.73 eV (BCN) to 2.72 eV (0.05-NVCN), 2.69 eV (0.15-NVCN), 2.67 eV (0.25-NVCN), and 2.63 eV (0.50-NVCN) with increasing KOH dosage, indicating that NVs effectively lower the bandgap and enhance light-harvesting capabilities. While BCN showed the lowest photocurrent density, introducing NVs increased photocurrent densities from 0.05-NVCN to 0.15-NVCN, attributed to improved electron excitation and migrations. However, excessive NVs in 0.25-NVCN and 0.50-NVCN reduced photocurrent density, likely due to structural degradation and the formation of recombination centers for photoexcited carriers.

When CVs were added to porous g-C_3_N_4_ nanosheets, various responses were observed, such as enhanced light absorption, a shift in the negative energy band, better-photogenerated electron capture, and molecular oxygen activation, which is helpful in reduction reactions [32]. Xu et al. [36] found that adding CVs to g-C_3_N_4_ improved the ability to absorb light in the 350–700 nm range. Light absorption across a wider range of wavelengths makes it easier for photogenerated electrons to move from the valence band (VB) to the conduction band (CB), making them more useful in reduction reactions [36]. According to the study of Preeyanghaa et al. [80], FCN has a moderate red shift at the absorption edge and an extended absorption tail of 450 to 800 nm compared to CN. The colors of as-synthesized catalysts range from light yellow to pale brown, directly reflecting its optical absorption capabilities. Slowly adding the formalin solution during synthesis reduced the band gap of catalysts from 2.94 eV (FCN) to 2.78 eV (CN). The band gap of FCN catalysts decreases because of the mid-gap condition in which the CVs develop. Han et al. [2] mentioned that creating CVs in g-C_3_N_4_ (Cv-CN) could produce electronic states below the conduction band (CB). This reduced band gap leads to enhanced light absorption. Cv-CN has a smaller energy gap and a lower conduction band potential than CN. Consequently, Cv-CN exhibits superior light absorption capabilities and effectively initiates photocatalytic reduction. It can be concluded that the enhanced light absorption depends on the bandgap. The bandgap depends on the density of vacancies present in the g-C_3_N_4_.

Changing the carbon defect density leads to variation in the bandgap. The progressive rise in formalin concentration reduced the bandgap (Eg) of FCN(10), FCN(20), and FCN(30) to 2.91 eV, 2.90 eV, and 2.78 eV, respectively, when compared to 2.94 eV for CN [80]. This reduction is related to mid-gap states caused by higher CV densities. Despite having the lowest Eg (2.78 eV), FCN(30) has reduced transient photocurrent responses and a higher interfacial charge transfer resistance than FCN(20). As a result, FCN(20) exhibits excellent photocatalytic degradation efficiency.

### 4.2. Increased Surface Area

Photocatalysts with a high specific surface area (SSA) and a porous structure are the most effective for boosting reactivity. The formation of enhanced SSA and pore size in g-C_3_N_4_ is due to the breakdown of multiple amino groups from the surface during a high-temperature hydrothermal reaction. Ammonia could function as a soft template to produce pores in CN. These results indicate that introducing vacancies to g-C_3_N_4_ may increase its SSA [50]. Liang et al. [116] noticed that the surface areas and nitrogen vacancy of the photocatalysts increased as the thermal treatment time increased. As a result, the surface area increased from 5.23 m^2^/g (for b-CN) to 57.65 m^2^/g (for nano-CN5), along with increased nitrogen vacancy. Li et al. [117] reported g-CN nanosheets with surface carbon vacancies (Cv-gCN) produced by a double thermal etching procedure under CO_2_ gas flow (using NaHCO_3_). NaHCO_3_ can produce CO_2_ gas in the atmosphere during calcination. The carbon vacancies and the porous structure form during the calcination process, leading to an increased surface area. Cv-gCN had a BET surface area of 147 m^2^ g^−1^, 5.4 times greater than g-CN (27 m^2^ g^−1^).

The improved surface area offers additional active sites, which is crucial for improving photocatalytic activity. Yang et al. [73] prepared thin-wall TCN g-C_3_N_4_ tubes by post-hydrothermal calcination in the presence of Ar gas, which exhibited a surface area of 34.37 m^2^/g. However, the carbon vacancy TCN (HTCN) produced from the calcination of TCN under hot vapor using Ar carrier gas exhibited a high SSA of 92.40 m^2^/g, which was 2.69 times higher than the TCN. The heptazine rings of g-C_3_N_4_ remain intact. The EPR spectra revealed a strong signal in the TCN, possibly due to local stress and bond angle disorder in the connective structure or unpaired electrons originating from carbon-atom sites. The decreased peak suggests that introducing carbon vacancies into the TCN enhances the specific surface area. Cai et al. [118] found many structural defects formed when g-C_3_N_4_ was calcined in a nitrogen environment using glyoxylic acid and its decomposition products. These flaws are susceptible to being eroded by oxidative etching. The presence of these high-density nitrogen vacancies results in a very porous structure, leading to an initial increase in the specific surface area with the addition of more glyoxylic acid (BET specific surface areas of GMNA-3 = 15.28 m^2^/g, GMNA-5 = 19.79 m^2^/g, GMNA-7 = 22.19 m^2^/g, and GMNA-10 = 114.95 m^2^/g). However, as the dose of glyoxylic acid increases, the specific surface area decreases (GMNA-15 = 86.16 m^2^/g), possibly because the pores collapse during the heating procedure. An excess amount of glyoxylic acid results in a high number of nitrogen vacancies and a decrease in the size of g-C_3_N_4_ after being heated in a nitrogen environment.

### 4.3. Influence on Charge Transfer and Migration

**Figure 23:**
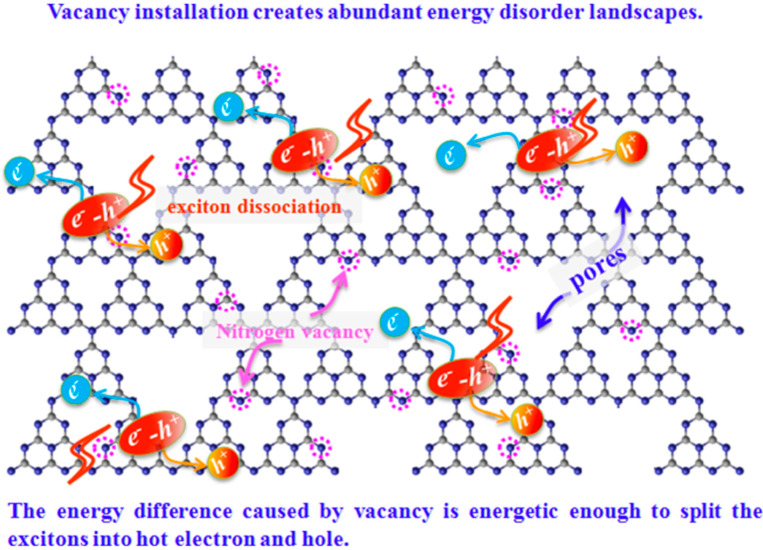
An illustration depicting a proposed mechanism for vacancy-mediated exciton dissociation in g-C_3_N_4_−VN Ns [119]. Circles mean the nitrogen vacancies.

Photocatalytic characteristics can be improved by altering the g-C_3_N_4_ morphology and structure and increasing carrier movement efficiency. Nowadays, developing diverse nanostructures is still an intriguing way to boost carrier transfer efficiency. Along with morphology, incorporating vacancies in the g-C_3_N_4_ can improve charge transfer and migration efficiency. Zhou et al. [119] illustrate the mechanism by which vacancies within g-C_3_N_4_-V_N_ nanosheets facilitate exciton dissociation (Figure 23). Adding g-C_3_N_4_ nanosheets with NVs could change the natural distribution of electronic states. It can form energy-disordered surfaces around the vacancies, with a bandgap energy difference of up to 0.35 eV compared to g-C_3_N_4_ nanosheets (i.e., g-C_3_N_4_ NS = 2.80 eV; NVs g-C_3_N_4_ NS = 2.45 eV). The exciton would split into free electrons and holes due to a high driving force greater than the exciton binding energy of 0.2 eV caused by the large energy difference. A significant energy difference (sufficient for hot-electron and hole creation) overcomes the strong Coulomb interactions between the electron and hole, demonstrating excellent electron-hole separation efficiency. As depicted in Scheme 1, the NVs vacancies act as sites that trap charge carriers, leading to reduced recombination of *e*^−^-*h*^+^ (electron–hole) pairs and thereby enhancing the overall photocatalytic activity of the material. With these benefits, the g-C_3_N_4_-V_N_ nanosheets exhibit exceptional photocatalytic efficacy in the reduction of organic contaminants such as 4-chlorophenol (4-CP), phenol (Ph), tetracycline (TET), and bisphenol A (BPA), compared to g-C_3_N_4_ nanosheets [119].

Photoluminescence (PL) spectra, transient photoelectric response (I-t), electrochemical impedance spectroscopy (EIS), and time-resolved fluorescence decay analysis have been used to study the e^−^ and h^+^ pairs’ recombination, separation and transfer, interfacial charge resistance, and migration, respectively. Researchers are achieving this by introducing an atom vacancy in g-C_3_N_4_ and doping and introducing vacancies. According to the study of Lv et al. [120], the insertion of O doping and N vacancies in CN results in improved photocatalytic hydrogen production. Based on the EIS analysis, they discovered that OCN-Nv (oxygen-doped CN with NVs) has the smallest arc radius, lower PL intensities, and enhanced photocurrent response compared to CN-Nv (CN-NVs), OCN (oxygen-doped CN), and CN. Introducing O and N vacancies in the CN enhances carrier separation and migration. Doped O induces spin polarization and changes the band structure, allowing charge separation in the bulk phase. The electrons then travel to the OCN-Nv surface, where N vacancies act as active sites for electron capture. These absorbed electrons are then transferred to adsorbed water molecules, resulting in a reduction reaction to produce H_2_. Han et al. found that adding carbon vacancies to the CN could improve the reduction of photogenerated electron and hole recombination [2]. Cv-CN had a higher saturation photocurrent density than CN. Comparably, the Nyquist plot’s smallest semicircle diameter denotes the lowest migration resistance among carriers. It results from the ability of CVs to function as sites for electron trapping, preventing photogenerated charges from recombination. More carriers can migrate to the photocatalyst surface in preparation for the subsequent redox reactions.

**Figure 24:**
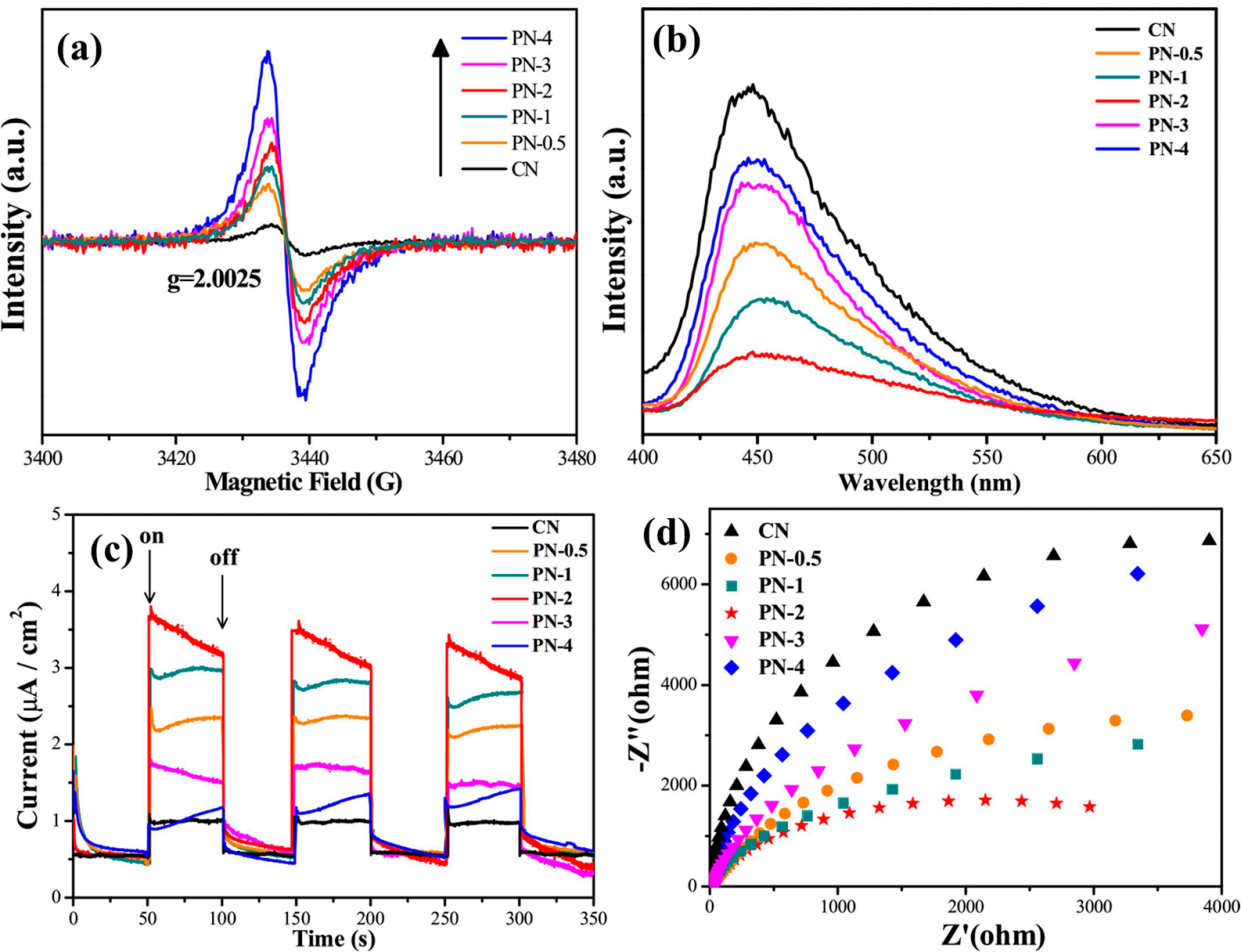
(**a**) EPR spectra; (**b**) photoluminescence spectra of CN and PN-*x*; (**c**) transient photocurrent; and (**d**) electrochemical impedance spectra of CN and PN-*x* [40].

Introducing CVs and NVs in the photocatalyst can improve charge separation and transfer efficiency by trapping the photogenerated charge carriers. For instance, Pang et al. [48] found that incorporating carbon and nitrogen vacancies in PCNS-F photocatalysts improved the efficiency of charge separation and transfer, surpassing that of BCN. PCNS-F photocatalysts demonstrated significantly increased photocurrent, reduced arc radius in EIS studies, decreased fluorescence intensity, and a shortened average lifetime of photogenerated carriers. These findings suggest improved charge separation, less resistance in electron transmission, reduced recombination, and faster migration and separation of charges. The reduced lifespan of photogenerated carriers may be attributed to quantum confinement effects and dual vacancy defects in carbon and nitrogen. Cao et al. [40] created a sequence of NVs containing g-C_3_N_4_/g-C_3_N_4_ *p-n* homojunction (PN-x) using secondary calcination at various intervals. Figure 24a shows a single Lorentzian line with a g value of roughly 2.0025; the number of NVs progressively increased as the secondary calcination time was prolonged. When they extend the secondary calcination period in the presence of H_2_ atmosphere, the EPR increases, confirming the creation of more unpaired electrons. However, the PL and I-t show that the intensity was initially lowered and then increased, as shown in Figure 24b,c. EIS spectra indicate that interfacial electron transfer resistance initially decreased but continued to rise as NV density increased (Figure 24d). This reduced charge separation, electron transport, and recombination data indicate that the high density of NVs may operate as recombination sites for electrons and holes. So, they obtained a better photocatalytic activity for just PN-2. It means that optimized NVs in g-C_3_N_4_ are necessary for better separation and less recombination, which is crucial for better photocatalytic activity.

### 4.4. Active Sites

Incorporating atom vacancies, particularly nitrogen vacancies, has been shown to significantly enhance its photocatalytic performance by facilitating charge separation and excitation processes, thereby increasing the overall efficiency of g-C_3_N_4_ in these applications. Additionally, introducing these vacancies not only improves the surface area and visible light absorption but also promotes the formation of active sites crucial for photocatalytic reactions, ultimately leading to enhanced interaction with reactants and improved charge dynamics within the material [121,122,123].

**Figure 25:**
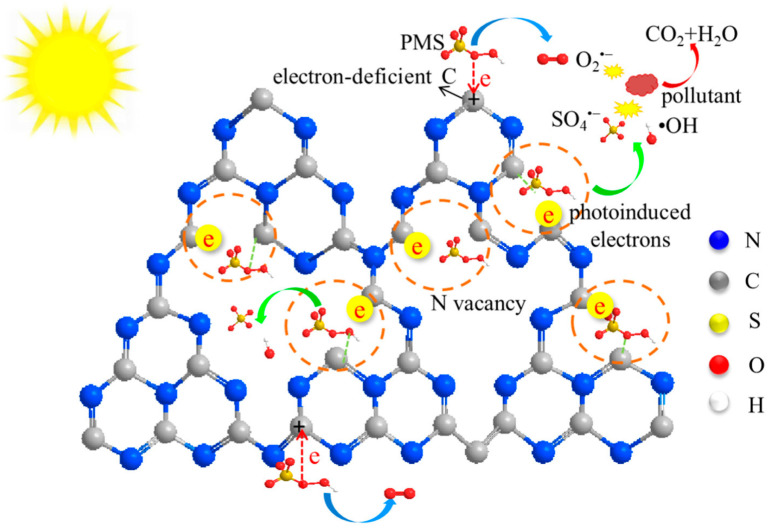
Schematic representation of PMS activation on dual active sites of VCN [125].

The inclusion of NVs in g-C_3_N_4_ increases additional active sites [124], Wang et al. [125] showed two different ways of breaking down or mineralization of peroxymonosulfate (PMS) using g-C_3_N_4_ nanotubes that have been modified with NVs vacancies. NVs vacancies in the VCN produce additional active sites around the NVs sites. As proposed in Figure 25, NV vacancies act as photogenerated electron trapping and adsorption sites. Transferring trapped electrons to PMS results in their reduction into SO_4_^•−^ and •OH. Meanwhile, electron-deficient C atoms pull the electrons from PMS due to the resulting NV vacancy. This electron transfer leads to the oxidation of PMS into O_2_^•−^ radicals.

**Figure 26:**
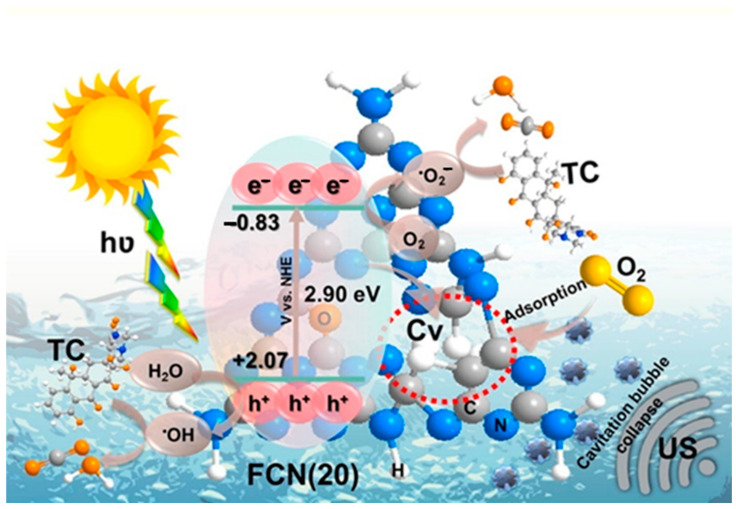
Schematic illustration depicting the degradation of Tetracycline in the presence of FCN [80].

Adding CV vacancies to g-C_3_N_4_ immediately increases the number of reactive active sites [126]. Preeyanghaa et al. [80] proposed a photocatalytic degradation of tetracycline using carbon vacancy FCN, as shown in Figure 26. According to their proposed strategy, the addition of CVs and self-doped oxygen sites in the FCN framework hindered e^−^/h^+^ pair recombination, enhanced molecular oxygen (O_2_) adsorption on CVs, and produced plenty of reactive species, resulting in the mineralization of tetracycline. The researcher has used ESR/EPR studies to demonstrate that CVs work as active or absorption sites. For instance, Dong et al. [126] found that the ESR peak intensity of Ns-g-C_3_N_4_ became equal to g-C_3_N_4_ after absorption of NO (the intensity of Ns-g-C_3_N_4_ peak is lower than the g-C_3_N_4_ before absorption of NO). It means that the CVs can adsorb NO by interacting with the O atom of NO and leaving the radical at the N-atom, which causes the ESR signal of the Ns-g-C_3_N_4_ sample to increase.

Summarized results are listed in Table 2 to compare the precursors, preparation methods, type of vacancy, morphology, bandgap, application, and efficiency of recently reported defect-mediated C_3_N_4_-based photocatalysts. It demonstrates the effects of reactant and synthesis parameters on the vacancy type, morphology, bandgap, and photocatalytic performance of PI-based and g-C_3_N_4_ photocatalysts.

## 5. Conclusions and Future Prospective

This review summarizes the planned creation of vacancy-based PI-polymer and g-C_3_N_4_ photocatalyst. We have explored the vacancies controlling by adjusting factors such as polycondensation temperature, reducing agent concentration, amount of doping agent, chemical vapor deposition, and etching time duration. We also discussed the synthesis techniques, including hydrothermal, calcination, thermal polymerization, plasma etching, chemical etching, gas etching, CVD, microwave-assisted CVD, and non-metal atom doping. We also discussed the important characterization techniques, such as EPR, XPS, XRD, NMR, FTIR, and PAS, which provide vital insights. We also summarized the importance and role of oxygen, carbon, and nitrogen vacancies. We are exploring the potential applications of these vacancy-based photocatalysts in environmental remediation and energy conversion, highlighting their significance in sustainable practices. We propose future research directions to optimize these vacancy-based systems for enhanced efficiency and sustainability in energy conversion processes.

Integrating oxygen-vacancy defects in polymer photocatalysts offers valuable insights into visible-light absorption and the adsorption and activation of molecular oxygen. However, research on oxygen-vacancy polymer photocatalysts still faces considerable limitations. A deeper understanding of these defects could drive the development of more efficient and sustainable photocatalytic materials. The research about how oxygen vacancies affect the catalytic properties of polymers can help researchers design innovative materials with enhanced photocatalytic performance. In addition to improving photocatalytic efficiency, understanding the fundamental mechanisms and the relationship between structure and function is equally important. Current research lacks a comprehensive understanding of the photocatalysis process and the mechanisms associated with oxygen-vacancy polymer photocatalysts, which are critical for the systematic design of high-performance materials. Advanced spectroscopy techniques and cutting-edge theoretical research are essential in this field. The following are important research topics for future study.

(1) Defects of conjugated polymer photocatalysts: Some reviewer papers report the defects of g-C_3_N_4_ photocatalysts. However, according to our literature review, the formation and effects of defects in conjugated polymer photocatalysts have not yet been reported in the review paper. In this review, we summarize the formation methods, characterization, and roles of vacancies in PI-based photocatalysts and g-C_3_N_4_ photocatalysts. Since there are few research papers investigating the defects of conjugated polymer photocatalysts, researchers can spend more effort on this topic in the future. Furthermore, despite the wide availability of amine and dianhydride monomers, only a small fraction of polyimides (PIs) has been investigated for photocatalytic applications. A high-throughput screening approach that combines experimental and computational methods could significantly accelerate the study of defective PI and other conjugated polymer photocatalysts;
(2) Key factors for reproducibility and stability: Despite the significant physicochemical stability of the PI polymer and g-C_3_N_4_, researchers frequently overlook the reproducibility and stability of the vacancy-modified PI polymer and g-C_3_N_4_ as crucial parameters for practical photocatalytic reactions. Furthermore, we have yet to investigate the key factors that influence this stability thoroughly. Therefore, future research should focus on these variables by conducting more comprehensive and in-depth experimental and theoretical analyses. In summary, the persistent efforts of global researchers indicate a promising and innovative future for PI polymers and g-C_3_N_4_-based nanomaterials;
(3) Effective characterization methods for finding the vacancy defects-activity correlation: Even with advances in defect engineering, significant challenges remain in forecasting the correlation between vacancy defects and the activity of modified g-C_3_N_4_ and conjugated polymer photocatalysts. Effective characterization methods and sophisticated calculation theories can explore such relationships and additional aspects of defect-rich g-C_3_N_4_. For instance, certain details, such as the location and concentration of the defect, as well as the distinction between surface and bulk defects, can be acquired through positron annihilation spectroscopy (PAS). Therefore, spatial distribution and concentration are essential aspects of defective g-C_3_N_4_ that warrant a more precise investigation;
(4) Optimized defect concentration: Although vacancies enhance the generation of e^−^/h^+^ pairs, introducing additional defects may still increase the likelihood of their recombination. Therefore, making the strategic design of vacancies is a vital consideration. Choosing a suitable synthesis technique is essential to effectively balance the electronic properties, electron–hole separation, and surface photoreactions. Future research should focus on finding the best conditions that balance the rates of e^−^/h^+^ generation and separation and other processes that slow the rate of progress. Furthermore, we must elucidate the relationship between stability, defect type, and specific effects. This challenge necessitates focusing on meticulous control, uniform dispersion and enhanced stability of defects to leverage their benefits for optimal performance;
(5) Environmentally benign and readily available modifiers: Consequently, it is anticipated that investigations will be conducted on environmentally benign and readily available modifiers, which can also be seamlessly integrated into the production of oxygen vacancy-conjugated polymers (PI) and vacancy-rich g-C_3_N_4_.

## Figures and Tables

**Table 1:** The advantages and disadvantages of various methods for the formation of vacancies in polymer photocatalysts.

Method	Advantages	Disadvantages	Reference
Calcination /Thermal polymerization	Scalable, straightforward synthesis, single vacancy creations	Constrained vacancy control and distributions, High temperatures required	[18]
Hydrothermal /Solvothermal	Fine-tuning of morphology, Minimal structural disruption	Prolonged reaction time	[55]
Chemical vapor deposition (CVD): Microwave-assisted CVD, Muffle furnace-assisted CVD	Easy synthesis, Uniform distribution of vacancies Time-saving	Expensive and challenging to scale for bulk materials	[60,61,62]
Non-metal atom doping	Improved stability, multifunctional photocatalyst	Complex synthesis	[63]
Chemical etching	High scalability, surface vacancies,	Structural damage, reducing stability	[69]
Gas etching	High scalability, most of the vacancies can be created on the surface, Precise vacancy control	Complex synthesis, expensive	[71,72]
Plasma treatment	Precise vacancy density control	Complex optimization and expansive	[77,78]

**Table 2:** The precursors, preparation methods, type of vacancy, morphology, bandgap, application, and efficiency of defect-mediated C_3_N_4_-based photocatalysts.

Precursors	Method	Vacancy Type	Morphology	Bandgap (eV)	Applications	Efficiency	Ref.
Melamine and sulfur	Calcination 550 °C	N vacancies	ultrathin sheet-like	2.76	H_2_ production	3.16 mmol g^−1^ h^−1^	[110]
Melamine	spark plasma sintering 550 °C	N vacancies	nanosheets	3.07	H_2_ production O_2_ production	1.51 mmol g^−1^ h^−1^ 0.75 mmol g^−1^ h^−1^	[88]
Melamine and (NH_4_)_2_CO_3_	calcination 550 °C	C vacancies	lumpy structures	2.80	H_2_ production	972.3 μmol g^−1^h^−1^	[35]
g-C_3_N_4_ (glycerol/H_2_O)	Solvothermal	N vacancies (N_3C_)	nanosheets	2.75	CO_2_ reduction	4.18 μmol g^−1^h^−1^ (CO)	[55]
Bulk g-C_3_N_4_	Hydrothermal followed by calcination at 500 °C	Dual or C and N vacancies	ultra-thin lamellar structure	2.63	Degradation of tetracycline and methylene blue	81.7% 97.7% 120 min	[48]
Molten urea	Formalin assisted calcination at 500 °C	C vacancies	sheet-like stacking structures	2.91	Degradation of tetracycline	97%, 60 min	[80]
Bulk g-C_3_N_4_	Ar atm, 550 °C	C vacancies	lamellar	2.78	H_2_O_2_ production metronidazole degradation	984 μmol L^−1^h^−1^ 90.7%, 100 min	[32]
Bulk g-C_3_N_4_	Etching with NaBH_4_	C vacancies	layered stacking	2.70	H_2_ production Cr(VI) reduction	1764.9 μmol g^−1^h^−1^	[36]
Ammonium acetate, urea	Calcination at 550 °C	N vacancies (N_3C_)	sheet-like structure	2.39	N_2_ fixation H_2_O_2_ production	1915 μmol h^−1^ g^−1^ 1098 μmol h^−1^ g^−1^	[90]
N vacancy g-C_3_N_4_	Hydrothermal at 180 °C	C and N vacancies	lamellar structure	2.45	Degradation of tetracycline	≥99.6%, 3 min	[56]
Bulk g-C_3_N_4_	In air, Calcination at 550 °C	C vacancies	wrinkled thin nanosheets	-	Degradation of Direct Red 227/direct Black 166	91.5%, 120 min 77.6%, 120 min	[38]
g-C_3_N_4_	In N_2_, Calcination at 550 °C	N vacancies	nano-belt	2.11	Degradation of propylparaben	94.3%, 30 min	[41]
Urea and KOH	Calcination at 550 °C	N vacancies	fragmented	2.46	H_2_O_2_ production	623.5 μmol g^−1^h^−1^	[91]
g-C_3_N_4_	etching Chromic acid solution	N vacancies	thinner nanolayers	2.79	H_2_ production CO_2_ reduction	2608 μmol g^−1^ h^−1^ 15.4 μmol g^−1^ h^−1^	[112]
Thin-wall tube g-C_3_N_4_	Calcination at 500 °C under 70 °C Vapor using Ar carrier gas	C vacancies	thin-wall tubes	2.94	H_2_ production	6588 μmol g^−1^ h^−1^	[73]
urea, thiourea, acetamide, Melamine	Calcination at 550 °C	N vacancies	nanosheets	2.96	degradation of atrazine (ATZ)	95.55%, 240 min	[43]
bulk g-C3N4	Calcination at 400 °C	C vacancies	lamellar structure	2.70	H_2_ production	1.69 mmol·g^−1^·h^−1^	[70]
Needle shaped g-C_3_N_4_	Ar atm, Calcination at 600 °C	N vacancies	porous hollow tubes	2.93	H_2_ production Degradation of Tetracycline and RhB	2028.9 μmol g^−1^ h^−1^ 79.5%, 10 min 100%, 10 min	[74]
Melamine, Urea, thiourea	Calcination (CVD)	S,O co-doped N vacancies	Nanosheets	2.85	Degradation of Methyl orange (MO)	~90%, 30 min	[44]
BCN	Hydrothermal etching with NH_4_F, calcination at 550 °C under Ar	F decorated C vacancies	2D sheet	2.99	ozonation of sodium p-perfluorinated nonoxybenzenesulfonate (OBS)	99.2%, 30 min	[64]
BCN Melamine	Calcination at 520 °C under Ar atm. Melamine-NH_4_Cl hydrothermal, and Calcination at 550 °C under N_2_	C vacancies N vacancies	sheet-like structure sheet-like structure	2.67 2.69	H_2_O_2_ Production H_2_O_2_ Production	187.9 μmol g^−1^·h^−1^ 159.0 μmol g^−1^·h^−1^	[2]
Melamine, Cyanuric acid and Sulfur	Solvothermal, calcination (200 °C/with sulfur, 550 °C).	N vacancies and S dopants	hollow nanobox	2.68	H_2_ production	4219.9 µmol g^−1^ h^−1^	[127]
Melamine (HCN)	550 °C (BCN) HCl Hydrothermal (HCN), NaBH_4_ calcination, N-defect	N vacancies	Nanosheets	2.27	H_2_O_2_ production	329.6 μmol h^−1^g^−1^	[128]
Melamine	Second calcination 650 °C	N vacancies	disordered pores with corrosion	1.78	H_2_ production	1102 µmol g^−1^ h^−1^	[129]
Thiocyanuric acid	calcination 550 °C	Sulfur doped and C, N vacancies	lamellar and ribbon-like structure	2.61	H_2_ production	17.70 mmol g^−1^ h^−1^	[130]
Melamine, boron solution and NH_4_Cl	calcination 550 °C	B doped N vacancies	Porous nanosheets	2.47	H_2_ production CO_2_ reduction	6959 µmol g^−1^ h^−1^ 69.1 µmol g^−1^ h^−1^ (CO)	[131]
Bulk g-C_3_N_4_ and Carbon Spheres	Hydrothermal 160 °C	N vacancies	regular spherical	2.40	CO_2_ reduction Cr(VI) reduction	7.68 μmol g^−1^h^−1^ (CO)	[132]
Melamine, ammonium iodide	Calcination at 500 °C	I-doped C vacancies	overlapped and stacked	2.45	Degradation of tetracycline	99.8 %, 150 min	[133]
Melamine, Cyanuric acid	Ar/H_2_ gas, Calcination at 500 °C	N vacancies	hollow microtubule	2.57	CO_2_ reduction	7.06 μmol g^−1^h^−1^ (CO)	[134]
g-C_3_N_4_	formic acid etching	N vacancies	irregular lamellar structures	2.74	H_2_ production CO_2_ reduction	1170 μmol g^−1^ h^−1^ 3.5 μmol g^−1^ h^−1^ (CO)	[135]
Melamine and cyanuric acid	Calcination at 520 °C	O-doped N vacancies	hollow nanotubes	2.83	H_2_ production RhB degradation	421.3 μmol g^−1^ h^−1^	[136]
BCN	etching with H_3_BO_3_ 500 °C under N_2_	B-doped N vacancies	Thin nanosheets	2.70	Degradation of Tetracycline	100%, 40 min	[137]
BCN	Treating with sodium bisulfite at RT	N vacancies	nanosheets	2.77	CO_2_ reduction Cr(VI) reduction	5.74 μmol·g^−1^·h^−1^ (CO), 1.30 μmol· g^−1^·h^−1^ (CH_4_)	[138]

## Data Availability

The data supporting the conclusions of this article will be made available on request.

## Abbreviations

OVs: Oxygen vacancies; NVs: Nitrogen vacancies; CVs: Carbon vacancies; CN: Carbon nitride: g-C_3_N_4_/g-CN; PMDE-MA: pyromellitic acid diethyl esters-melamine salt monomer; R-PI: OVs rich polyimide; D-PI: OVs deficient polyimide; PTA-MA: perylene tetracarboxylic acid-melamine crystals; PTA-MA-Tn: OVs perylene tetracarboxylic acid-melamine synthesized at different polycondensation temperatures; PI-NaHCO_3_: OVs rich polyimide-NaHCO_3_; PCN-F: NVs polymeric carbon nitride freeze-drying; PCN-T: NVs polymeric carbon nitride dried at 65 °C; PCNS-T: Dual vacancy polymeric carbon nitride obtained from dried at 65 °C PCN-T; PCNS-F: Dual vacancy polymeric carbon nitride obtained from freeze-dried PCN-F; SCNNS: CVs sulfur doped carbon nitride nano sheets; CNNR: NVs carbon nitride nanoribbons; VO-CN: OVs carbon nitride; FCN-Cv: Fluorine-coated carbon nitride CVs; S-PDCN: Sulfur doped CVs carbon nitride; N_3_C vacancies: Nitrogen connected with 3 carbons (NVs position); TCN: Carbon nitride tubes; N-(C)_3_: Nitrogen connected with 3 carbons; CN-C_V2_: Carbon nitride CVs prepared using 1.5 g of sulfamic acid; PCN: polymeric carbon nitride; PCN-50: polymeric carbon nitride-temperature (50 °C); HCN: Carbon nitride synthesized using H_2_O; ICN: Graphitic carbon nitride synthesized using isopropanol; HTCN: Ultrathin porous g-C_3_N_4_ tubes with Nb vacancies; TCN: Graphitic carbon nitride tubes; PHCN: Conical porous hollow carbon nitride; HCN: Hexagonal tubes g-C_3_N_4_; 3NBCN: 3 represents the concentration of NaBH_4_ used for synthesis of CVs in the g-CN; GCN: Graphitic CN; BCN: Bulk CN; FCN-Cv: Fluorine coated CN-CVs; HPHT: High pressure and high temperature; PNCN-2: Porous NVs g-C_3_N_4_ prepared at 2GPa/300 °C using HPHT apparatus; 50CN: NVs g-CN prepared using 50 mL of glycerol and 10 mL of water; SPS: Spark plasma sintering; hCN: high crystallinity g-C_3_N_4_; BCN400: Boron doped NVs g-C_3_N_4_ 400 °C (obtained at 400 °C calcination of mixture of g-C_3_N_4_ and NaBH_4_); CN–B: Boron doped g-C_3_N_4_; CN–H: Nitrogen deficient g-C_3_N_4_; NCN 0.48: NVs g-C_3_N_4_ prepared using mixture of 0.48 g of sulfamic acid and 1.0 g of melamine; PCN: Primitive g-C_3_N_4_; CH_3_CO_2_NH_4_: Ammonium acetate; g-C_3_N_4_-N_3_C: g-C_3_N_4_ with three-coordinate nitrogen (N_3_C) vacancies; g-C_3_N_4_-N_3_C-X: g-C_3_N_4_ with three-coordinate nitrogen (N_3_C) vacancies-Conc. of CH_3_CO_2_NH_4_; g-C_3_N_4_-N_3_C-0.5: g-C_3_N_4_ with three-coordinate nitrogen (N_3_C) vacancies- 0.5g of CH_3_CO_2_NH_4_; Nv-M: g-C_3_N_4_ with rich NVs and cyano groups; Cv-PCNNS-550: CVs porous g-C_3_N_4_ nanosheets prepared at 550 °C; FCN (20): 20 uL of formalin solution assisted CVs g-C_3_N_4_; CN_V_-mCN-Br: Br-doped C, N-deficient g-C_3_N_4_; ODH-CNX: oxalyl dihydrazide- g-C_3_N_4_ (X = concentration of ODH); ODH-CN2: 0.2 g of ODH-g-C_3_N_4_ (mixture of 0.2 g ODH and 20 g Urea); g-C_3_N_4_-V: surface CVs g-C_3_N_4_; Cv-g-C_3_N_4_: CVs g-C_3_N_4_; N-(C)_3_: Three coordinated nitrogen; N-(C)_2_: Two coordinated nitrogen; GMNA: Glyoxylic acid and melamine calcination in the presence of N_2_ gas and secondary calcination in the presence of air atmosphere; GMNA-no (3/5/7/10/15): Represents the melamine percentage to glyoxylic acid molar ratios.
